# Toward Flexible Printed Electronics: A Spider‐Silk‐Inspired, Strong and Tough Thermoplastic Polyamide Elastomer

**DOI:** 10.1002/advs.75129

**Published:** 2026-04-02

**Authors:** Bo Yang, Liyue Zhang, Mengjing Yang, Hangzhi Guo, Zhibo Lin, Xinyu Qi, Wenjun Li, Haiyan Yan, Meng Li, Yanhui Chen, Weixing Chen, Zhenguo Liu, Wei Huang

**Affiliations:** ^1^ Institute of Flexible Electronics Northwestern Polytechnical University Xi'an China; ^2^ Key Laboratory of Flexible Electronics of Zhejiang Province Ningbo Institute of Northwestern Polytechnical University Ningbo China; ^3^ School of Flexible Electronics (SoFE) and Henan Institute of Flexible Electronics (HIFE) Henan University Zhengzhou China; ^4^ Engineering Research Center of Light Stabilizers for Polymer Materials Universities of Shaanxi Province School of Materials and Chemical Engineering Xi'an Technological University Xi'an China

**Keywords:** flexible printed electronics, hierarchical structure, hydrogen bond, thermoplastic polyamide elastomer

## Abstract

The advancement of intelligent and compliant flexible electronic devices demands polymer substrate materials that exhibit both high strength and toughness, which are critical for the performance and durability of printed circuits. Inspired by the structure of spider silk, we designed and synthesized a series of naphthalene‐containing thermoplastic polyamide elastomers (TPAE) in this work. The optimized TPAE‐*N_40%_*‐*PEG_25%_* exhibits an impressive hydrogen bonding density of up to 41.8%, leading to outstanding mechanical properties: a tensile strength of 37.3 MPa, an elongation at break of 470.8%, and a remarkable toughness of 134.6 MJ/m^3^. When it was employed as a flexible substrate film (0.1 mm thickness), it reliably supported printed conductive traces, demonstrating stable electrical signal output under cyclic deformation. Furthermore, the substrate attached to fingers and wrists successfully monitored bending motions through consistent electrical responses, highlighting its potential for flexible strain sensors. Notably, the printed circuits can be completely removed by soaking in anhydrous ethanol for 12 h, enabling the full recovery and reuse of the substrate film. This spider‐silk‐inspired strategy provides a new design pathway for developing strong, tough, and sustainable polymer substrates for next‐generation flexible electronics.

## Graphical abstract

Inspired by spider silk, a bio‐inspired polyamide elastomer with a dynamic physical cross‐linking network is developed. Mimicking the rigid β‐sheet crystals and sacrificial hydrogen bonds of silk, the material achieves an exceptional balance of strength and toughness. Applied as a flexible printed electronic substrate, it demonstrates stable circuit performance and enables convenient recycling by ethanol washing.

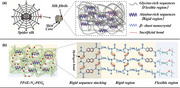

## 1 Introduction

Flexible printed electronics (FPE) is an advanced approach for fabricating electronic devices based on printing technologies, with its core involving the deposition of conductive functional materials onto flexible substrates [1, 2, 3, 4]. This technology combines the advantages of printing, such as large‐area, low‐cost, and compatible flexible processing, with the lightweight, thin, flexible, and transparent characteristics of organic/polymeric functional materials used as substrates [5, 6, 7, 8]. Consequently, FPE demonstrates broad application prospects in fields including wearable devices, healthcare, smart textiles, and automotive electronics [9, 10, 11, 12]. As the carrier of FPE, the properties of the substrate directly determine the reliability and application boundaries of the final device. An ideal flexible printed substrate possesses excellent mechanical durability to withstand repeated bending, folding, and even rolling, alongside good thermal stability to endure post‐processing treatments (e.g., thermal curing) without significant dimensional changes, shrinkage, deformation, or decomposition [13, 14, 15, 16]. Commonly used flexible substrates are primarily categorized into elastomers and polymer films. Among elastomers, thermoplastic polyurethane (TPU) has been widely adopted as substrate materials due to their outstanding elasticity, toughness, and processability [17, 18]. However, the limited heat resistance of most TPU severely restricts their application with noble metal electronic pastes that require higher temperature curing (> 100°C) [19, 20, 21]. Furthermore, some synthesis routes for TPU rely on organic solvents, posing additional environmental concerns [22]. While polymer films like polyethylene terephthalate (PET) and polycarbonate (PC) offer transparency and excellent impact resistance as flexible printing substrates, their mechanical durability is often insufficient, particularly prone to failure under dynamic bending conditions [23]. Polyimide (PI) films, despite being ultrathin and highly flexible, suffer from inherent brittleness and lack of elasticity as their major drawbacks when used as flexible substrates, coupled with high cost being another limiting factor for their widespread adoption [24]. Consequently, traditional polymer substrates, such as elastomeric TPU and plastic films like PET, PC, and PI, often exhibit a trade‐off in performance: they are either constrained by insufficient thermal stability, plagued by poor dynamic fatigue resistance, or lack considerations for eco‐friendly recycling. This inherent difficulty in simultaneously achieving high dynamic fatigue resistance, thermal stability, and recyclability has become a critical bottleneck restricting the advancement of flexible printed electronics toward high‐performance and sustainable development. The development of a novel substrate capable of transcending the aforementioned performance trade‐off is therefore urgently required.

**SCHEME 1:**
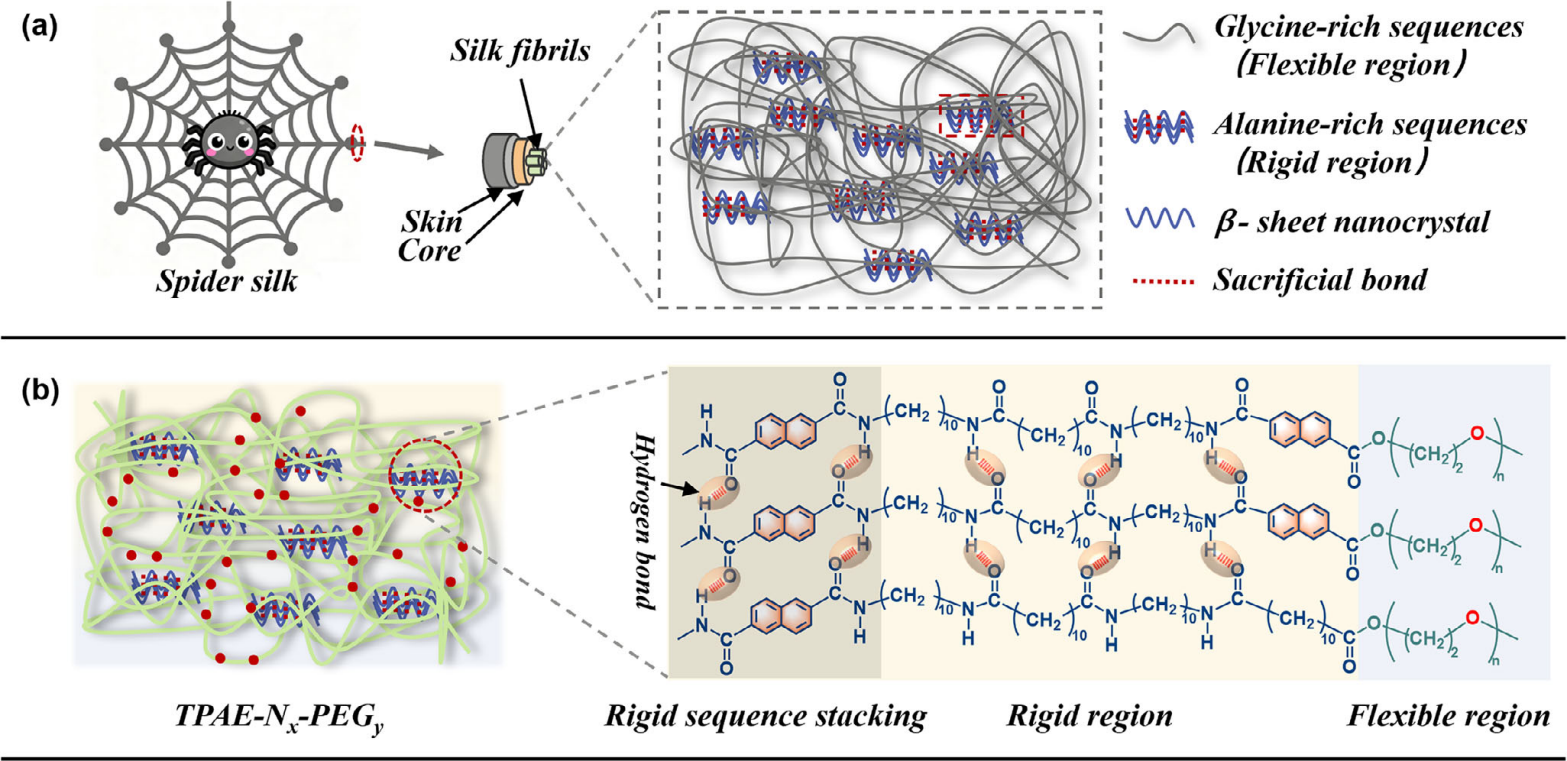
(a) The multi‐level structure and dynamic sacrificing bonds of spider silk. (b) The polymer structure of *TPAE‐N_x_‐PEG_y_*.

In nature, spider silk achieves an ideal combination of strength and toughness, precisely meeting the performance requirements of flexible printed electronic substrates. This outstanding mechanical property stems from its intricate hierarchical microstructure and dynamic sacrificial bonds (Scheme 1a) [25, 26]. The primary structure of spider silk proteins features alternating rigid alanine‐rich sequences (which form β‐sheet nanocrystals) and flexible glycine‐rich sequences, providing the molecular basis for microphase separation [27]. These β‐sheet crystals act as physical cross‐linking points, providing immense strength, while the hydrogen‐bond networks between them function as dynamic sacrificial bonds that reversibly break and reform under stress, effectively dissipating energy and conferring high toughness [28]. This energy dissipation mechanism, arising from the reversible rupture and reconstruction of the dynamic hydrogen‐bond network, effectively prevents stress concentration and inhibits microcrack propagation under cyclic mechanical loading, thereby endowing spider silk with exceptional dynamic fatigue resistance at the macroscopic level. This rigid‐flexible nanoscale composite structure is key to spider silk's balance of strength and toughness. This delicate balance of rigid and flexible components offers a compelling blueprint for designing synthetic polymer systems with superior mechanical performance.

Inspired by this natural design, introducing similar biomimetic principles into the molecular design of polymer—by constructing a multi‐level dynamic physical cross‐linking network—holds promise for developing a novel flexible printed electronic substrate polymer that surpasses the performance of traditional elastomers. Thermoplastic polyamide elastomers (TPAE), a class of high‐performance polymer consisting of polyamide hard segments and polyether soft segments, have garnered significant attention in recent years [29, 30]. TPAE combines remarkable resistance to high and low temperatures (−50°C to 200°C), exceptional mechanical strength, and rubber‐like flexibility, making them ideal candidate materials for flexible substrates in printed electronics [31, 32, 33, 34]. The comprehensive properties of TPAE originate from their unique microphase‐separated structure: the crystalline polyamide hard segments contribute high tensile strength, tear resistance, and impact resistance, outperforming conventional general‐purpose elastomers and resisting wear or cracking even under dynamic stress; Meanwhile, the polyether soft segments impart excellent low‐temperature flexibility and elasticity [35, 36, 37]. Furthermore, by precisely tuning the chemical structure, ratio, and molecular weight of the hard and soft segments, the material properties can be tailored on demand [38]. The synthesis of TPAE predominantly employs an environmentally friendly melt polycondensation process, which avoids the use of organic solvents and aligns with sustainable development requirements [39].

Inspired by the multi‐level structure and sacrificial bond mechanism of spider silk, a series of strong and tough naphthalene‐containing polyamide elastomers was designed and synthesized via melt polycondensation in this work. As illustrated in Scheme 1b, the molecular structure comprises polyamide (PA1012) hard segments derived from 1,10‐diaminodecane (DA10) and 1,12‐dodecanedioic acid (DDA), polyethylene glycol (PEG) soft segments, and 2,6‐naphthalenedicarboxylic acid (NDA)—a unit featuring a rigid *π–π* conjugated planar structure—incorporated to modulate the polymer's hierarchical structure and dynamic sacrificial bonds. The alternating copolymerization of crystalline PA1012 segments (rigid blocks) and ether‐bond‐containing PEG (flexible blocks) forms a primary structure analogous to spider silk protein, providing the molecular basis for microphase separation. The constructed amide‐naphthalene‐amide rigid sequence within the chains, coupled with the synergistic effect of *π–π* stacking of naphthalene rings and hydrogen bonding between amide groups, forms physical cross‐linking points [40]. This creates a secondary structure reminiscent of the β‐sheet crystals in spider silk, contributing strength, thermal stability, and resistance to molecular chain slippage in polyamide elastomer. Furthermore, the inherent dynamic reversibility of the intermolecular hydrogen bonds enables them to break and re‐form under stress, providing a mechanism for energy dissipation and enhanced toughness, thereby achieving a balance between strength and toughness [41, 42, 43]. The hard segment domains are dispersed within the soft segment matrix, forming a nanoscale “island‐sea” morphology, ultimately leading to an excellent balance of strength and toughness in the material. Finally, we applied this strong and tough polyamide elastomer inspired by spider silk to the flexible electronic printing substrate film. Conductive pathways were printed onto this film using electronic paste. The resulting circuits exhibited stable electrical signal responses under various conditions. Moreover, the flexible printed substrate could be recycled and reused by immersing it in anhydrous ethanol and wiping off the printed circuits. We believe that this spider‐silk‐inspired strategy for preparing strong and tough thermoplastic elastomers offers a promising substrate material solution for the future large‐scale commercialization of sustainable flexible electronic systems.

## 2 Results and Discussion

### 2.1 Structural Characterization and Molecular Weight Determination

**FIGURE 1:**
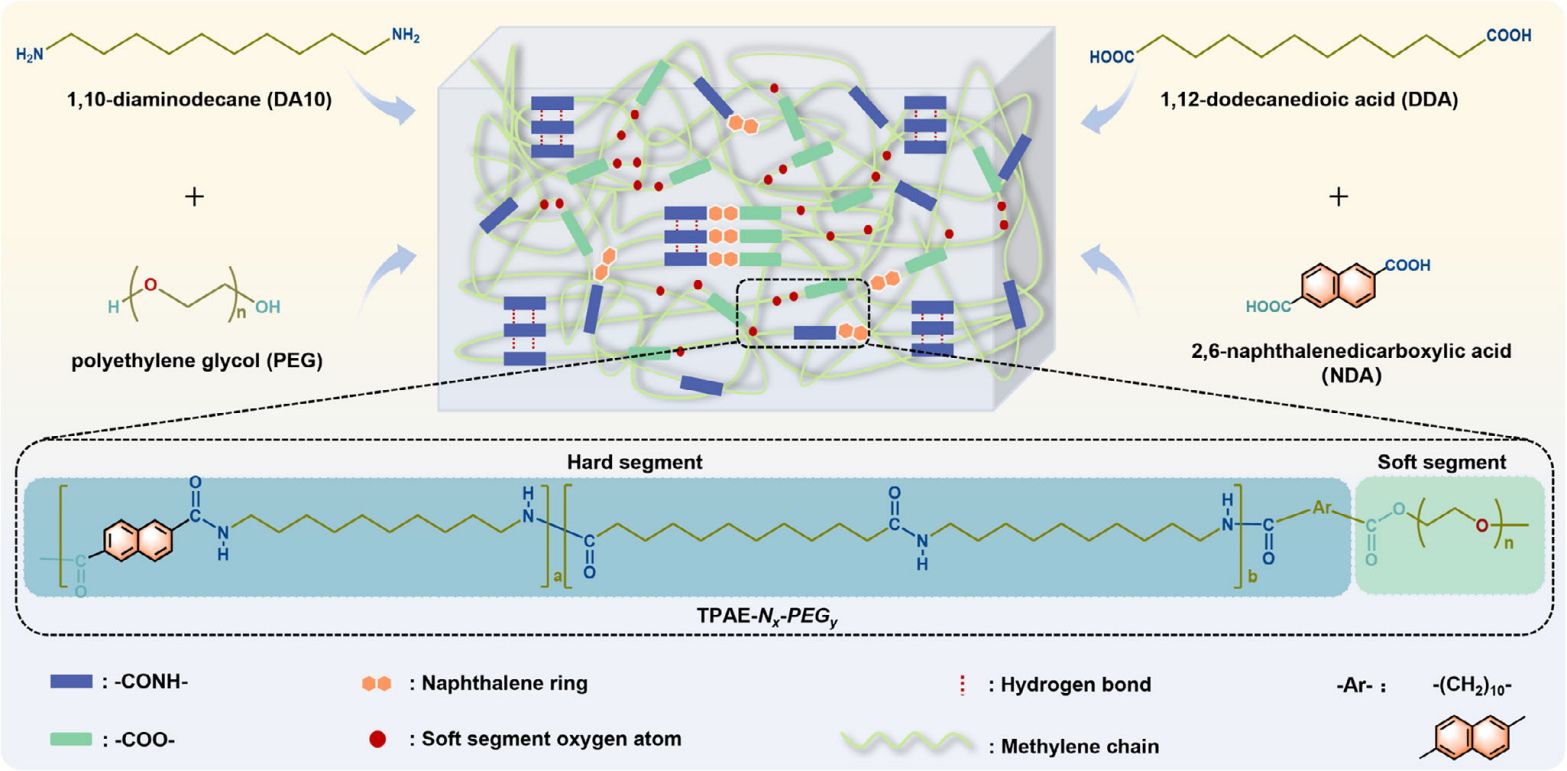
The synthetic route of TPAE‐*N_x_*‐*PEG_y_*.

The synthetic route of naphthalene‐containing polyamide elastomer is shown in Figure 1. The NDA, DDA, DA10, and PEG‐400 were added into a 2 L high‐temperature and high‐pressure reactor in accordance with the molar ratio of monomers as shown in Table S1 for the melt polycondensation reaction. The final product was named TPAE*‐N_x_‐PEG_y_*, where *N* and *PEG* represent NDA and PEG‐400, respectively, and *x* and *y* represent the mole percentage contents of NDA and PEG‐400 in the feedstock. For example, in TPAE*‐N_40%_‐PEG_25%_*, *x* = 40% indicates that the content of NDA in the feedstock is 40 mol%, and *y* = 25% indicates that the content of PEG‐400 in the feedstock is 25 mol%. To confirm the successful synthesis of TPAE‐*N_x_*‐*PEG_y_*, the structural characterization, inherent viscosity number ([*η*_int_]), and molecular weight determination were conducted.

**FIGURE 2:**
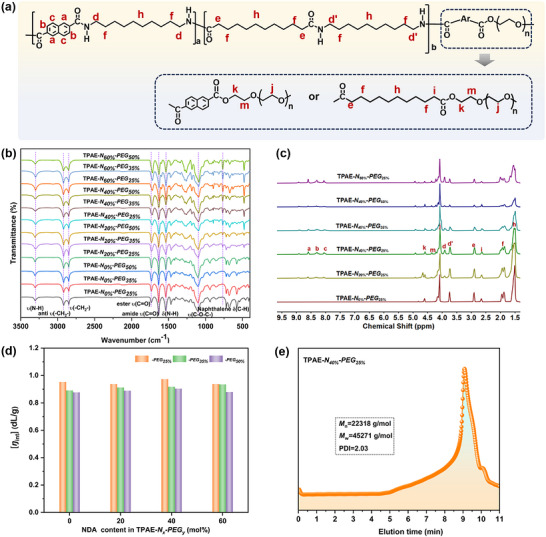
(a) The molecular chain structure formula of TPAE‐*N_x_*‐*PEG_y_*. (b) FT‐IR spectrum of TPAE‐*N_x_*‐*PEG_y_*. (c) ^1^H NMR spectra of TPAE‐*N_x_*‐*PEG_y_*. (d) The [*η*_int_] of TPAE‐*N_x_*‐*PEG_y_*. (e) The molecular weight of TPAE‐*N_40%_*‐*PEG_25%_* was tested by GPC.

The molecular chain structure formula of TPAE‐*N_x_*‐*PEG_y_* was shown in Figure 2a. The Fourier Transform Infrared Spectroscopy (FTIR) of TPAE‐*N_x_*‐*PEG_y_* is presented in Figure 2b. The peak at 3302 cm^−1^ corresponds to the N‐H stretching vibration of the amide bond. The peaks at 2919 cm^−1^ and 2849 cm^−1^ are attributed to the stretching vibration of methylene groups on the carbon chain. The peak at 1732 cm^−1^ corresponds to the C═O stretching vibration of the ester group [34]. This suggests the occurrence of an esterification reaction, likely between carboxyl groups from DDA or NDA and hydroxyl groups from PEG‐400, leading to ester bond formation. The peak at 1634 cm^−1^ is associated to the C═O stretching vibration of the amide bond (amide I). The peak at 1537 cm^−1^ corresponds to the N‐H bending vibration coupled with C─N stretching in the amide bond (amide II) [40]. This indicates the presence of amide bonds in the polymer structure, confirming the formation of hard segments in TPAE‐*N_x_*‐*PEG_y_*. The peak at 1103 cm^−1^ corresponds to C─O─C stretching vibrations in the TPAE‐*N_x_*‐*PEG_y_* soft segments [34]. This indicates the PEG‐400 block structure was introduced in TPAE‐*N_x_*‐*PEG_y_* macromolecular. The out‐of‐plane C─H bending vibration of naphthalene rings in TPAE‐*N_x_*‐*PEG_y_* appears at 768 cm^−1^. Based on the above, the structure of TPAE‐*N_x_*‐*PEG_y_* was initially determined.

To further confirm the structure of TPAE‐*N_x_*‐*PEG_y_*, the proton nuclear magnetic resonance (^1^H NMR) spectrum of TPAE‐*N_x_*‐*PEG_y_* is presented in Figure 2c. In this figure, the letters representing proton hydrogen are the same as those in Figure 2a. The chemical shifts at 8.43 ppm (position a), 8.13 ppm (position b), and 7.90 ppm (position c) are assigned to the protons on the naphthalene ring. The chemical shifts at 3.85 ppm (position d) and 3.74 ppm (position d’) correspond to the methylene protons on the DA10 near the amide bond [40]. This change indicates amide bond formation between the carboxyl and amino groups, resulting in an altered proton chemical shift in DA10. The chemical shift at 2.90 ppm (position e) is assigned to the methylene protons near the amide bond on the DDA. The chemical shift at 1.88 ppm (position f) is assigned to the methylene protons in the region near the amide bond on the TPAE‐*N_x_*‐*PEG_y_*. The chemical shift near 1.51 ppm (position h) is assigned to the methylene protons farther from the amide bond on the TPAE‐*N_x_*‐*PEG_y_*. The chemical shift at 2.65 ppm (position i) is assigned to the methylene protons near the ester group [34]. The chemical shift at 4.08 ppm (position j) is assigned to the methylene protons on the soft segment chain. The chemical shift at 4.61 ppm (position k) and 4.20 ppm (position m) is assigned to the methylene protons on the soft segment chain adjacent to the ester group. This indicates that a bond‐forming reaction occurred between the soft and hard segments, yielding an ester linkage, resulting in a change in the proton chemical shift of PEG‐400. Based on the above, the structure of TPAE‐*N_x_*‐*PEG_y_* was further confirmed by ^1^H NMR spectrum.

The [*η*_int_] of TPAE‐*N_x_*‐*PEG_y_* was calculated using the one‐point method (Equation ). The relative viscosity (*η*_r_) and increased viscosity (*η*_sp_) of TPAE‐*N_x_*‐*PEG_y_* were obtained by using Equations S1 and S2. The *C* of Equation 1 represents the concentration (1 g/dL) of the solution. The calculation results were presented in Figure 2d and Table S2. These results indicate that the [*η*_int_] of TPAE‐*N_x_*‐*PEG_y_* polymers ranges from 0.88 dL/g to 0.97 dL/g, consistent with the expected viscosity range for TPAE [34]. Interestingly, the [*η*_int_] of TPAE‐*N_x_*‐*PEG_y_* progressively decreased with increasing PEG‐400 content incorporated into the molecular chain. This phenomenon is potentially attributable to a reduction in the reactivity between hydroxyl and carboxyl groups as the molecular chain extends with higher PEG‐400 content. Furthermore, the molecular weight and molecular weight distribution (polydispersity index, PDI) of TPAE‐*N_40%_*‐*PEG_25%_* were determined using gel permeation chromatography (GPC). The mobile phase was hexafluoroisopropanol (HFIP) at a temperature of 35°C and a flow rate of 1 mL/min. The GPC results, shown in Figure 2e, revealed a number‐average molecular weight (*M*_n_) of 22 318 g/mol, a weight‐average molecular weight (*M*_w_) of 45 271 g/mol, and a PDI of 2.03 for TPAE‐*N_40%_*‐*PEG_25%_*. The results obtained from both the one‐point calculation method and GPC analysis confirm the TPAE‐*N_x_*‐*PEG_y_* with the anticipated [*η*_int_] and high molecular weights.

(1) $$\left[\eta_{\mathrm{int}}\right]={\left[2\left(\eta_{sp}-\ln\eta_{r}\right)\right]}^{\frac{1}{2}}/C$$

### 2.2 Thermal Property Analysis of TPAE*‐N_x_‐PEG_y_*

**FIGURE 3:**
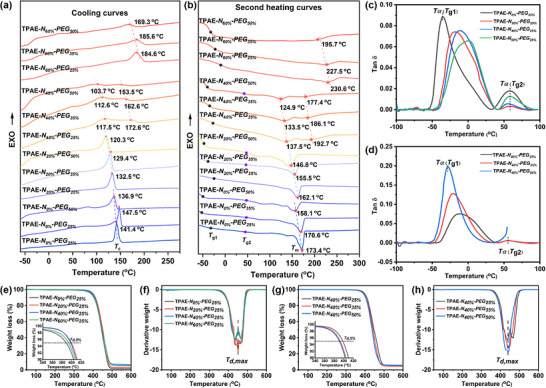
(a) The cooling DSC curves of TPAE‐*N_x_*‐*PEG_y_*. (b) The second heating DSC curves of TPAE‐*N_x_*‐*PEG_y_*. (c) The loss factor (Tan δ) of TPAE‐*N_x_*‐*PEG_25%_*. (d) The Tan δ of TPAE‐*N_40%_*‐*PEG_y_*. (e) TGA curves of TPAE‐*N_x_*‐*PEG_25%_*. (f) DTG curves of TPAE‐*N_x_*‐*PEG_25%_*. (g) TGA curves of TPAE‐*N_40%_*‐*PEG_y_*. (h) DTG curves of TPAE‐*N_40%_*‐*PEG_y_*.

The melting and crystallization processes of TPAE‐*N_x_*‐*PEG_y_* as a function of temperature were investigated using Differential Scanning Calorimetry (DSC). The melting temperature (*T*_m_) and crystallization temperature (*T*_c_) were summarized in Table S3. Figure 3a shows the DSC cooling curves of TPAE‐*N_x_*‐*PEG_y_* after erasing thermal history. The *T*_c_ of TPAE‐*N_0%_*‐*PEG_25%_* was 141.4°C, whereas it decreased to 136.9°C for TPAE‐*N_0%_*‐*PEG_50%_* when the PEG‐400 content increased to 50 mol%. This indicates that incorporating the soft segment (PEG‐400) into TPAE‐*N_x_*‐*PEG_y_* influences the crystallization behavior of the polymer, thereby affecting its *T*_c_. Analysis of the cooling curves reveals that under constant hard segment conditions, the *T*_c_ of TPAE‐*N_x_*‐*PEG_y_* decreases monotonically with increasing molar fraction of the soft segment in the molecular chain. However, upon the introduction of naphthalene rings into the molecular chain, the *T*_c_ of TPAE‐*N_x_*‐*PEG_y_* exhibits a non‐monotonic trend, initially decreasing and subsequently increasing. This behavior may be attributed to an ordered–disordered–ordered transition within the molecular chain as the comonomer (NDA) content increases. The initial ordered state arises because the system, without NDA, constitutes a ternary copolymer. The relatively simple composition facilitates crystallization of the hard segments in TPAE‐*N_0%_*‐*PEG_y_*, resulting in a higher *T*_c_. Introducing 20 mol% NDA disrupts the crystallization state of the DDA segments. The molecular chain regularity of the hard segments decreases, and co‐crystallization between different chain segments becomes difficult. Consequently, the *T*_c_ of TPAE‐*N_20%_*‐*PEG_25%_* (132.5°C) is lower than that of TPAE‐*N_0%_*‐*PEG_25%_* (141.4°C), as observed in the DSC cooling curves. When the NDA content increases to 40 mol%, the DSC cooling curves of TPAE‐*N_40%_*‐*PEG_y_* reveal double crystallization peaks. At this composition, crystallization of the NDA‐containing segments competes with that of the DDA‐containing segments, resulting in a disordered molecular chain arrangement in TPAE‐*N_40%_*‐*PEG_y_*. Upon further increasing the NDA content to 60 mol%, the crystallization peak in the DSC cooling curves of TPAE‐*N_60%_*‐*PEG_y_* reverts to a single peak. This suggests that crystallization of the hard segments becomes dominated by NDA‐containing segments. Furthermore, the planar conjugated naphthalene ring, together with its terminal amide groups, forms a rigid amide‐naphthalene‐amide sequence that facilitates ordered stacking and promotes hard‐segment crystallization. The DSC second heating curves of TPAE‐*N_x_*‐*PEG_y_* shown in Figure 3b follow the same trend as the cooling curves. Notably, the *T_m_* of TPAE‐*N_60%_*‐*PEG_25%_* reaches 230.6°C, representing an increase of 57.2°C compared to the *T_m_* of TPAE‐*N_0%_*‐*PEG_25%_* (173.4°C). This significant enhancement is ascribed to the incorporation of naphthalene rings, which form the amide‐naphthalene‐amide long rigid sequences. The synergistic effects of hydrogen bonding between the amide groups and the planar conjugated naphthalene ring endow TPAE‐*N_x_*‐*PEG_y_* with superior thermal stability upon heating.

To investigate the relationships of molecular chain mobility of TPAE‐*N_x_*‐*PEG_y_* and temperature, Dynamic Thermomechanical Analysis (DMA) was employed in this work. The peak temperature of the α‐transition observed in the polymer molecular chains was defined as the glass transition temperature (*T*_g_) of TPAE‐*N_x_*‐*PEG_y_*, with specific data provided in Table S3. Figure 3c illustrates the variation in *T*_g_ of TPAE‐*N_x_*‐*PEG_25%_* as a function of NDA content in the molecular chain, while maintaining a constant soft segment content of 25 mol%. The loss factor (Tan δ) curves of TPAE‐*N_x_*‐*PEG_25%_* exhibit two distinct peaks within the temperature range of −100°C to 100°C, indicating the presence of two characteristic temperatures associated with the activation of polymer chain segment mobility. The peak located below room temperature (25°C) corresponds to the mobilization of the polyether‐based soft segments in TPAE‐*N_x_*‐*PEG_25%_*, denoted as *T*_g1_. This behavior is attributed to the structural characteristics of the soft segment repeat units: the C─O bond possesses low energy and exhibits facile rotation, while the ether oxygen atom reduces steric hindrance from adjacent groups. This combination acts as an internal “lubricant” for the molecular chains, resulting in an extremely low energy barrier for internal rotation. Consequently, the chain segments can initiate mobility at relatively low temperatures. The peak observed above room temperature (25°C) corresponds to the mobilization of the amide‐based hard segments in TPAE‐*N_x_*‐*PEG_25%_*, denoted as *T*_g2_. When the system temperature reaches *T*_g2_, the polyamide hard segments acquire sufficient energy for coordinated motion, marking the transition of the polymer from glassy state to rubbery state. As the NDA content in the TPAE‐*N_x_*‐*PEG_25%_* molecular chain increases from 0 mol% to 60 mol%, *T*_g1_ rises from −35.9°C to 1.7°C. This indicates that the incorporation of NDA, which constructs rigid amide‐naphthalene‐amide structures within the molecular chain, imposes constraints on the temperature‐dependent responsive mechanisms of the soft segments. This further demonstrates that in TPAE‐*N_x_*‐*PEG_25%_*, despite the thermodynamic drive for microphase separation between soft and hard segments, extensive molecular‐level interactions and mutual constraints persist between them. In contrast, *T*_g2_ of TPAE‐*N_x_*‐*PEG_25%_* remains constant at approximately 57.7°C. This stability suggests that the glass transition of the hard segment domains is predominantly governed by the internal hydrogen‐bonding network and crystallinity. Furthermore, the peak intensity of *T*_g2_ is significantly lower than that of *T*_g1_, indicating that the hard segment portion of TPAE‐*N_x_*‐*PEG_25%_* exhibits a lower Tan δ value. This is characteristic of superior elastic recovery performance and reduced internal energy dissipation, primarily conferred by the stable physical cross‐linking network of the hard segments and the well‐defined microphase‐separated structure within the material. Figure 3d explores the variation in *T*_g_ of TPAE*‐N_40%_‐PEG_y_* as a function of soft segment content, while the NDA content in the hard segments was fixed at 40 mol%. It was observed that as the soft segment content increased from 25 to 50 mol%, the *T*_g1_ of TPAE*‐N_40%_‐PEG_y_* decreased from −12.1°C to −28.0°C. This indicates that increasing the soft segment content further promotes the formation of a purer soft segment amorphous phase in TPAE*‐N_40%_‐PEG_y_*, thereby significantly lowering *T*_g1_. This enhancement in soft phase mobility allows the material to retain its elastomeric properties at lower temperatures, effectively broadening its low‐temperature application window.

To investigate the thermal decomposition conditions of TPAE‐*N_x_*‐*PEG_y_*, Thermogravimetric analysis (TGA) was employed, and the detailed data were provided in Table S3. From Figure 3e, it was found that the initial decomposition temperature (*T_d,5%_*) of TPAE‐*N_0%_*‐*PEG_25%_* was 389.0°C. The introduction of NDA into the molecular chain resulted in a slight increase in *T_d,5%_*. The maximum decomposition rate temperature (*T_d,max_*) of TPAE‐*N_x_*‐*PEG_25%_* exhibited multiple peaks in Figure 3f, indicating differences in the decomposition rates among different molecular chain segments. Specifically, the ether‐containing soft segments in TPAE‐*N_x_*‐*PEG_25%_* decomposed more rapidly at lower temperatures, whereas the polyamide hard segments decomposed at higher temperatures. As illustrated in Figure 3g, for TPAE‐*N_40%_*‐*PEG_y_*, the *T_d,5%_* decreased from 398.1°C to 391.0°C as the soft segment content increased from 25 mol% to 50 mol%. Furthermore, with the soft segment content increasing, the *T_d,max_* of TPAE‐*N_40%_*‐*PEG_y_* gradually shifted toward lower temperatures, as shown in Figure 3h. In summary, the incorporation of NDA into the polyamide hard segments of TPAE‐*N_x_*‐*PEG_y_* enhances both *T_d,5%_* and *T_d,max_*, while increasing the soft segment content promotes a shift of *T_d,5%_* and *T_d,max_* to lower temperatures.

### 2.3 Crystallization Properties of TPAE*‐N_x_‐PEG_y_*

**FIGURE 4:**
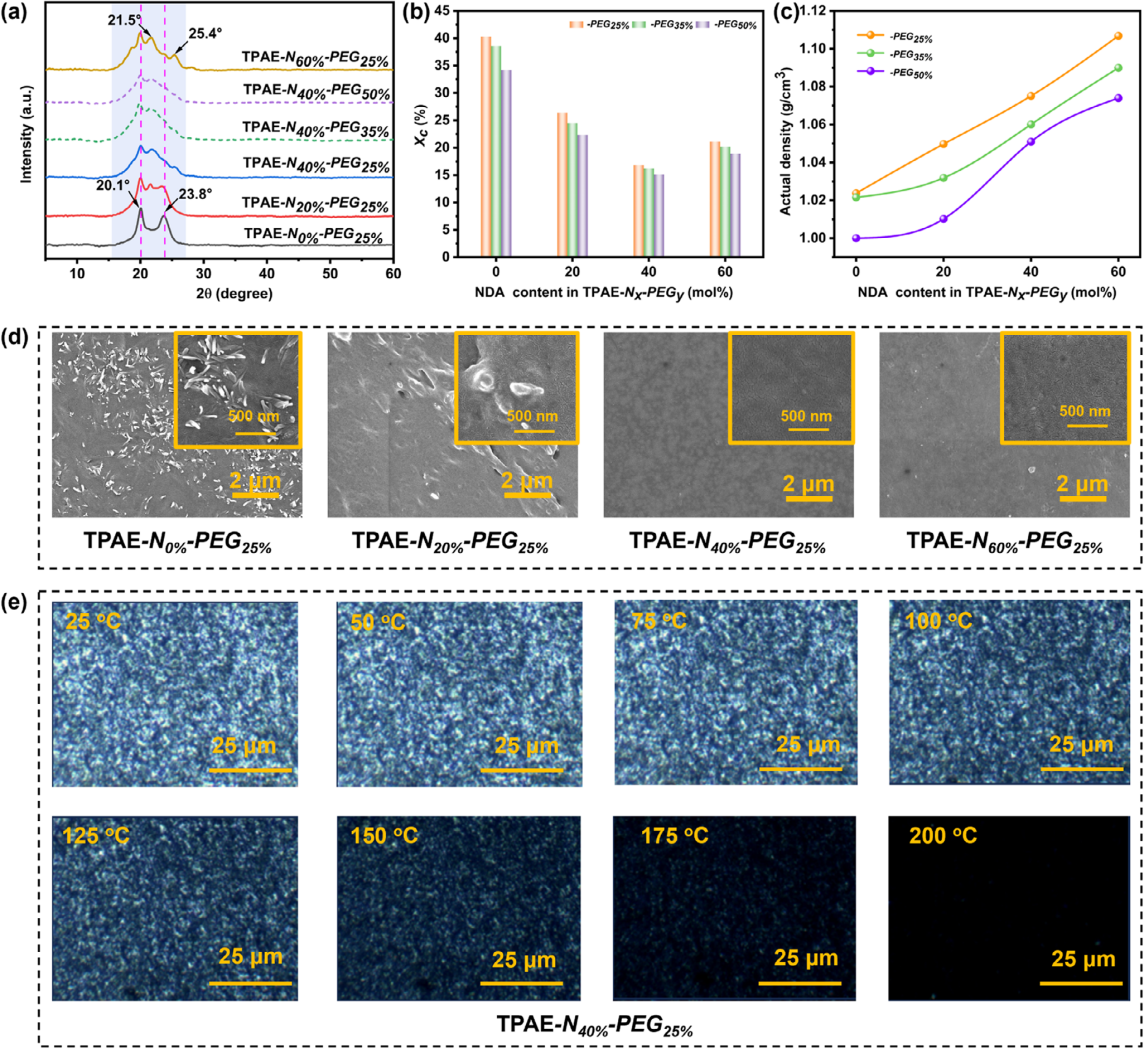
(a) WAXD patterns of TPAE‐*N_x_*‐*PEG_y_*. (b) The *X_c_* of TPAE‐*N_x_*‐*PEG_y_*. (c) The actual density of TPAE‐*N_x_*‐*PEG_y_*. (d) The SEM images of TPAE‐*N_x_*‐*PEG_25%_*. (e) The POM images of TPAE‐*N_40%_*‐*PEG_25%_* with temperature.

The crystalline properties of TPAE‐*N_x_*‐*PEG_y_* were investigated by Wide‐Angle X‐ray Diffraction (WAXD), and the results are shown in Figure 4a. All WAXD curves exhibited diffraction peaks of varying intensities, indicating that TPAE‐*N_x_*‐*PEG_y_* is a typical semi‐crystalline polymer. When without NDA was incorporated into the molecular chain, TPAE‐*N_0%_*‐*PEG_25%_* showed two sharp diffraction peaks at 20.1°and 23.8°, suggesting the formation of well‐developed crystals. As the NDA content gradually increases, new diffraction peaks emerged at 21.5° and 25.4° in the WAXD curves, indicating the formation of new crystalline regions originating from NDA‐containing segments in TPAE‐*N_x_*‐*PEG_y_*. Meanwhile, with the NDA content increasing, the diffraction peaks at 21.5° and 25.4° became progressively stronger compared to those at 20.1° and 23.8°, further confirming the development of new crystalline structures based on NDA segments. When the NDA content was fixed at 40 mol%, and the soft segment content in TPAE‐*N_40%_*‐*PEG_y_* was increased from 25 mol% to 50 mol%, the diffraction peak at 25.4° decreased significantly, indicating that the content of soft segments influences the crystallization of hard segments. To further elucidate the influence of NDA and PEG‐400 content within the molecular chains on the crystalline properties of the polymers, the crystallinity (*X_c_*) of TPAE‐*N_x_*‐*PEG_y_* was calculated by integrating the WAXD curves and applying Equation S3. The results were presented in Figure 4b. The *X_c_* values of TPAE‐*N_x_*‐*PEG_y_* consistently exhibited a decreasing trend with increasing PEG‐400 content in the molecular chains, once again corroborating the above viewpoints that soft segments reduce the crystallinity of the polymer. Meanwhile, when the PEG‐400 content in TPAE‐*N_x_*‐*PEG_y_* was maintained at 25 mol%, the *X_c_* decreased from 40.3% to 16.8% as the NDA content in the molecular chains increased from 0 to 40 mol%. The primary reason is that the gradual increase of NDA content reduces the structural regularity of the polyamide hard segment molecular chains, leading to the decline in the crystallinity of TPAE‐*N_x_*‐*PEG_y_*. However, when the NDA content reached 60 mol%, the *X_c_* of TPAE‐*N_60%_*‐*PEG_25%_* increased to 21.1%, which is 4.3% higher than that of TPAE‐*N_40%_*‐*PEG_25%_*. This indicates that when the content of NDA exceeds that of DDA, the hard chain segments formed by NDA, due to their conjugated plane structure, exhibit higher structural regularity, and therefore their crystallinity is also higher than that of the hard chain segments formed by DDA. Meanwhile, the crystallinity and molecular chain composition of TPAE‐*N_x_*‐*PEG_y_* also affect its actual density. As shown in Figure 4c, for a constant NDA content, the actual density of TPAE‐*N_x_*‐*PEG_y_* exhibited a decreasing trend with increasing soft segment content within the molecular chains. This observed decrease in actual density is attributed to the reduction in the crystallinity of TPAE‐*N_x_*‐*PEG_y_* induced by the ether‐containing soft segments. For instance, at NDA content of 0 mol%, the actual density of TPAE‐*N_0%_*‐*PEG_y_* decreased from 1.02 to 1.00 g/cm^3^ as the soft segment content increased from 25 mol% to 50 mol%. Conversely, the actual density of TPAE‐*N_x_*‐*PEG_y_* increased with a higher molar ratio of NDA in the molecular chains. For example, at a constant soft segment content of 25 mol%, the actual density of TPAE‐*N_x_*‐*PEG_25%_* increased from 1.02 to 1.11 g/cm^3^ with increasing NDA content. This increase in density is attributed to the incorporation of the amide‐naphthalene‐amide structure into the molecular chains, which promotes greater molecular chain regularity and facilitates more orderly packing. This results in an increased packing density, thereby manifesting as an increase in the true density of the TPAE‐*N_x_*‐*PEG_y_*.

The crystalline morphology and microphase‐separated structure of TPAE‐*N_x_*‐*PEG_y_* were investigated using scanning electron microscopy (SEM). As shown in Figure 4d, in the SEM image of TPAE‐*N_x_*‐*PEG_25%_*, the bright regions correspond to hard domains, while the dark regions represent soft domains. The hard domains originate from the regular arrangement and crystallization of polyamide hard segments, whereas the soft domains consist of disordered polyamide segments and polyether soft segments. In the absence of NDA, TPAE‐*N_0%_*‐*PEG_25%_* exhibits a well‐ordered polyamide hard segment structure, which facilitates orderly packing and crystallization, leading to densely distributed hard domains in the SEM image. With the introduction of 20 mol% NDA (TPAE‐*N_20%_*‐*PEG_25%_*), the incorporation of amide‐naphthalene‐amide motifs disrupts the regularity of the hard segments, resulting in the crystallizability reducing. This is manifested as smaller and more dispersed hard domains. When the NDA content is increased to 40 mol% (TPAE‐*N_40%_*‐*PEG_25%_*), no distinct bright regions are observed in the SEM image. Instead, a homogeneous alternating contrast between light and dark areas appears, indicating a highly disordered state with no significant crystalline regions. Notably, at 60 mol% NDA (TPAE‐*N_60%_*‐*PEG_25%_*), the polyamide hard segments regain a tendency toward orderly arrangement, and distinct bright domains reappear, suggesting that a higher content of amide‐naphthalene‐amide units promotes the formation of a new ordered structure in the hard segments. Except for TPAE‐*N_40%_*‐*PEG_25%_*, all samples exhibit clear microphase‐separated morphologies under SEM. To further clarify the phase structure of TPAE‐*N_40%_*‐*PEG_25%_*, variable‐temperature polarized optical microscopy (POM) was employed to characterize its surface morphology (Figure 4e). At 25°C, the POM images show distinct bright and dark contrast, indicating the presence of microphase separation. However, the crystalline regions formed by the polyamide hard segments are small and uniformly dispersed within the amorphous matrix. As the temperature increases from 25°C to 200°C, the POM images gradually darken, suggesting that the crystalline hard segments melt and transition into an amorphous state, further confirming the existence of a microphase‐separated structure in this system.

### 2.4 Analysis of Hydrogen Bonds of TPAE*‐N_x_‐PEG_y_*

**FIGURE 5:**
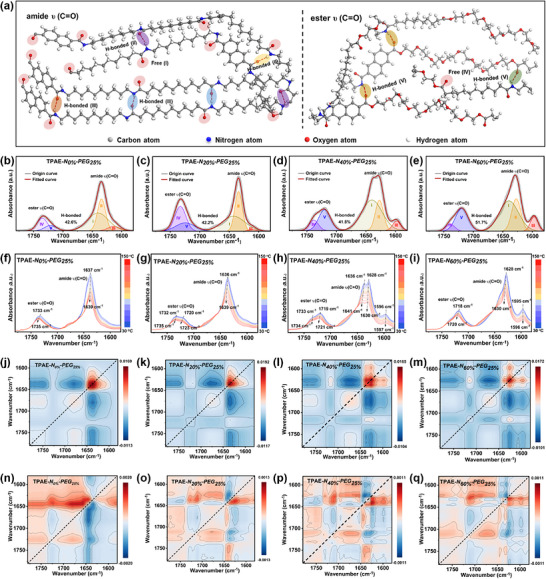
(a) The existence state of C═O in TPAE‐*N_x_*‐*PEG_y_*. FTIR spectra of (b) TPAE‐*N_0%_*‐*PEG_25%_*, (c) TPAE‐*N_20%_*‐*PEG_25%_*, (d) TPAE‐*N_40%_*‐*PEG_25%_*, (e) TPAE‐*N_60%_*‐*PEG_25%_* in the C═O stretching vibration region. VT‐FTIR spectra of (f) TPAE‐*N_0%_*‐*PEG_25%_*, (g) TPAE‐*N_20%_*‐*PEG_25%_*, (h) TPAE‐*N_40%_*‐*PEG_25%_*, (i) TPAE‐*N_60%_*‐*PEG_25%_* upon heating from 30°C to 150°C in the range of 1580–1780 cm^−1^. The 2D‐COS synchronous map of (j) TPAE‐*N_0%_*‐*PEG_25%_*, (k) TPAE‐*N_20%_*‐*PEG_25%_*, (l) TPAE‐*N_40%_*‐*PEG_25%_*, (m) TPAE‐*N_60%_*‐*PEG_25%_* under heating condition from 30°C to 150°C in the range of 1580–1780 cm^−1^. The 2D‐COS asynchronous map of (n) TPAE‐*N_0%_*‐*PEG_25%_*, (o) TPAE‐*N_20%_*‐*PEG_25%_*, (p) TPAE‐*N_40%_*‐*PEG_25%_*, (q) TPAE‐*N_60%_*‐*PEG_25%_* under heating condition from 30°C to 150°C in the range of 1580–1780 cm^−1^.

In TPAE*‐N_x_‐PEG_y_*, a large number of carbonyl (C═O) groups are present, and their states are closely related to the properties of the polymer, as shown in Figure 5a. In the polyamide hard segment chains, the C═O groups exist mainly in three states: free state (Free (I)), hydrogen‐bonded in amorphous regions (H‐bonded (II)), and hydrogen‐bonded in crystalline regions (H‐bonded (III)). In the ester linkages formed between soft and hard segments via polycondensation, the C═O groups exhibit two states: free state (Free (IV)) and hydrogen‐bonded state (H‐bonded (V)). By performing peak deconvolution on the FTIR spectra in the C═O stretching vibration region (1580–1780 cm^−1^), the relative content of hydrogen‐bonded C═O groups in TPAE*‐N_x_‐PEG_y_* with different NDA contents was quantitatively analyzed. The results are presented in Figure 5b–e and Table S4. When the NDA content increased from 0 to 40 mol%, the proportion of hydrogen‐bonded C═O decreased from 42.6% to 41.5%. This is mainly attributed to the disruption of molecular chain ordering by the incorporation of a small amount of NDA, which weakened the hydrogen‐bonding ability of C═O groups, leading to an increase in the proportion of free C═O (Free (I)) in the hard segment from 42.5% to 51.3%. However, when the NDA content further increased to 60 mol%, the proportion of hydrogen‐bonded C═O in TPAE*‐N_60%_‐PEG_25%_* increased to 51.7%. At this point, the polyamide chain segments are dominated by NDA units, and the planar conjugated structure of the naphthalene ring promotes ordered packing of the molecular chains, thereby facilitating the hydrogen bond formation. Meanwhile, as the NDA content increased from 0 to 60 mol%, the C═O stretching vibration peaks of the amide groups exhibited a redshift. Taking TPAE*‐N_x_‐PEG_25%_* as an example, the peak position of hydrogen‐bonded C═O in amorphous regions (H‐bonded (II)) shifted from 1634 to 1627 cm^−^^1^, while that in crystalline regions (H‐bonded (III)) shifted from 1613 to 1597 cm^−^^1^. This redshift indicates a decrease in the force constant of the C═O bond and a reduction in vibrational frequency, which can be attributed to the formation of an extended conjugated system between the naphthalene ring of NDA and the amide group (─CO─NH─), leading to electron delocalization. Together with the synergistic effect of intermolecular hydrogen bonding, these factors reduce the bond strength. As also indicated in Table S4, the proportion of hydrogen‐bonded C═O in ester groups (H‐bonded (V)) increased significantly from 7.2% to 21.5% with increasing NDA content, suggesting that the planar conjugated structure of the naphthalene ring also promotes hydrogen bonding at the soft–hard segment interface. Variable‐temperature FTIR (VT‐FTIR) was employed to study the changes in the C═O vibration peaks of TPAE*‐N_x_‐PEG_25%_* during heating from 30°C to 150°C at a rate of 10°C/min. The results are shown in Figure 5f–i. As the temperature increased, enhanced thermal motion of the molecular chains disrupted the originally ordered hydrogen‐bonding network, causing partial dissociation of hydrogen bonds. Consequently, some originally hydrogen‐bonded C═O groups transitioned to the free state, resulting in a blueshift of the C═O stretching vibration peaks. To further clarify the response of different types of hydrogen bonds during heating, 2D correlation spectroscopy (2D‐COS) was applied to analyze the VT‐FTIR spectra in the C═O region (1580–1780 cm^−^^1^). In the synchronous spectra, positive cross‐peaks (red) indicate that the signals change in the same direction. As shown in Figure 5j–m, distinct auto peaks were observed near the diagonal in the synchronous maps, located at 1597–1613 cm^−^^1^, 1627–1634 cm^−^^1^, 1639–1642 cm^−^^1^, 1720–1724 cm^−^^1^, and 1732–1739 cm^−^^1^, corresponding to the different states of C═O groups in amide and ester linkages. The asynchronous spectra (Figure 5n–q) were used to determine the sequence of vibrational responses. Taking TPAE*‐N_40%_‐PEG_25%_* as an example, by comparing the signs of cross‐peaks in the synchronous and asynchronous spectra (Tables S5–S7) and applying Noda's rules, the sequence of spectral responses upon heating was derived as follows: 1598 cm^−^^1^→1720 cm^−^^1^→1738 cm^−^^1^→1640 cm^−^^1^→1627 cm^−^^1^. This sequence indicates that hydrogen bonds in crystalline regions dissociate first, while those in amorphous regions are the most stable.

Figure S1a–c presents the changes in the state of C═O groups and the percentage of hydrogen bonding in the polymers with increasing soft segment content, while the NDA content was fixed at 40 mol%. The curve fitting results are summarized in Table S8. As the soft segment content increased from 25 to 35 mol%, the proportion of hydrogen‐bonded carbonyl groups in TPAE*‐N_40%_‐PEG_y_* decreased from 41.8% to 36.5%. This reduction can be attributed to the decreased ratio of hard segments in the polymer, which in turn reduced the extent of hydrogen bonding between carbonyl groups in the amide motifs. Specifically, when the soft segment content increased from 25 to 35 mol%, the proportion of disordered hydrogen bonds (II) in TPAE*‐N_40%_‐PEG_y_* decreased from 18.0% to 8.9%, and that of ordered hydrogen bonds (III) decreased from 5.5% to 2.4%. However, when the soft segment content reached 50 mol%, the proportion of hydrogen‐bonded carbonyl groups in TPAE*‐N_40%_‐PEG_50%_* increased to 45.8%. This is due to the reaction between most carboxyl groups in the polyamide hard segments and hydroxyl groups in the soft segments, leading to the formation of ester linkages. As a result, the hydrogen bonds associated with ester carbonyl groups (V) increased significantly from 18.3% in TPAE‐N40%‐PEG25% to 33.7%. VT‐FTIR spectroscopy was employed to characterize the temperature‐dependent changes of the C═O stretching bands (1580–1780 cm^−^^1^) of TPAE*‐N_40%_‐PEG_y_* at a heating rate of 10°C/min from 30°C to 150°C, as shown in Figure S1d–f. With temperature increasing, the carbonyl peaks shifted toward higher wavenumbers, a trend consistent with the results presented in Figure 5f–i. Based on the analysis of the 2D synchronous correlation spectra (Figure S1g–i, Table S9) and asynchronous correlation spectra (Figure S1j–l, Table S10) of TPAE*‐N_40%_‐PEG_y_*, the sequence of spectral changes corresponding to the dynamic processes of the five characteristic peaks was interpreted according to Noda's rules and the assignments provided in Table S11. The response order was inferred as follows: 1738 cm^−1^→1598 cm^−1^→1720 cm^−1^→1640 cm^−1^→1627 cm^−1^.

### 2.5 Mechanics Property Analysis

**FIGURE 6:**
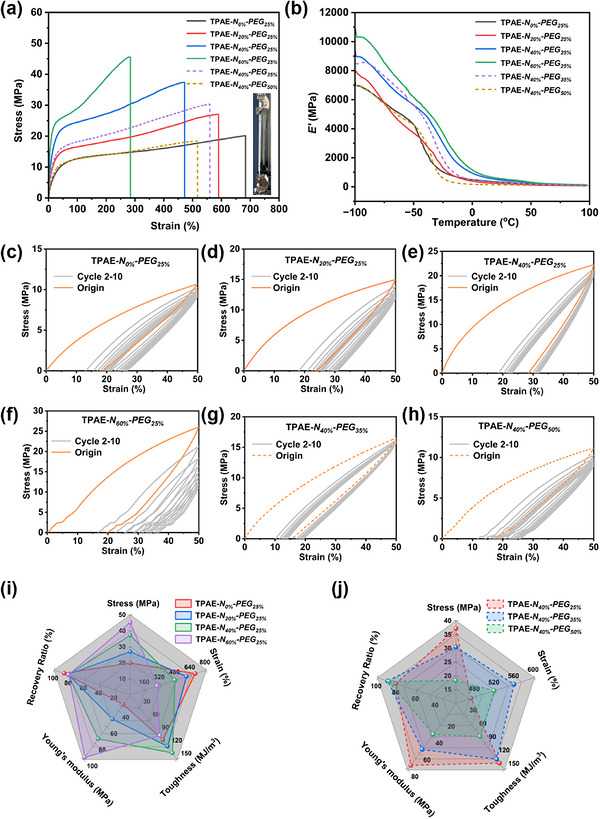
(a) The stress–strain curves of TPAE*‐N_x_‐PEG_y_*. (b) The *E’* of TPAE*‐N_x_‐PEG_y_*. The recovery curves of (c) TPAE*‐N_0%_‐PEG_25%_*, (d) TPAE*‐N_20%_‐PEG_25%_*, (e) TPAE*‐N_40%_‐PEG_25%_*, (f) TPAE*‐N_60%_‐PEG_25%_*, (g) TPAE*‐N_40%_‐PEG_35%_* and (h) TPAE*‐N_40%_‐PEG_50%_* stretching cycle 10 times. (i) Summary of TPAE*‐N_x_‐PEG_25%_* mechanical properties. (j) Summary of TPAE*‐N_40%_‐PEG_y_* mechanical properties.

The stress–strain curves of TPAE‐*N_x_*‐*PEG_y_* are shown in Figure 6a. When the soft segment content was 25 mol%, the stress of TPAE‐*N_x_*‐*PEG_25%_* increased with the NDA proportion rising. As the NDA content increased from 0 to 60 mol%, the fracture stress increased from 20.3 to 45.5 MPa, representing an enhancement of more than 100%. This strengthening effect can be attributed to the formation of long rigid amide‐naphthalene‐amide sequences along the molecular chains, coupled with intermolecular hydrogen bonding through amide linkages, which collectively improved the tensile strength of the polymer. However, the fracture strain of TPAE‐*N_x_*‐*PEG_25%_* decreased from 683.8% to 283.8% as the NDA content increased from 0 to 60 mol%, owing to the increased chain rigidity resulting from the higher content of rigid segments, which reduced the stretchability of the polymer. With the NDA content fixed at 40 mol%, increasing the soft segment proportion from 25 mol% to 35 mol% led to an increase in the fracture strain of TPAE‐*N_40%_*‐*PEG_y_* from 470.8% to 560.9%, while the fracture stress decreased from 37.3 to 30.3 MPa. The higher soft segment content improved the flexibility and extensibility of the molecular chains, but reduced the relative content of the rigid amide‐naphthalene‐amide segments, thereby lowering the tensile strength. When the soft segment content was further increased from 35 to 50 mol%, the fracture strain decreased from 560.9% to 515.5%, and the fracture stress declined from 30.3 to 18.2 MPa. When the content of the soft segment reaches 50 mol%, the condensation reaction between hydroxyl and carboxyl groups in the system significantly increases. However, due to the fact that the equilibrium constant of this reaction is much lower than that of the reaction between amino and carboxyl groups (less than 1% of it), the molecular weight of TPAE‐*N_40%_*‐*PEG_50%_* decreases, which in turn affects its mechanical properties [44]. In addition, chain entanglement and shielding effects in longer molecular chains significantly reduced the probability of end‐group encounters, hindering chain extension. The variation of the storage modulus (*E’*) of TPAE‐*N_x_*‐*PEG_y_* with temperature is presented in Figure 6b. When the soft segment content was maintained at 25 mol%, *E’* increased with higher naphthalene ring content in the molecular chains. This increase resulted from the greater proportion of rigid amide‐naphthalene‐amide structures and enhanced hydrogen bonding, which together contributed to a higher E’. In contrast, for samples with 40 mol% NDA content, the E’ of TPAE‐*N_40%_*‐*PEG_y_* decreased as the soft segment proportion increased.

Figure 6c–f presents the elastic recovery performance of TPAE‐*N_x_*‐*PEG_25%_* with different NDA contents after 10 cyclic tests at 50% strain. It can be observed that all TPAE‐*N_x_*‐*PEG_25%_* samples exhibit certain elastic recovery capability. Among them, TPAE‐*N_40%_*‐*PEG_25%_* demonstrates the most stable elastic recovery behavior after 10 cycles. To further investigate the influence of polymer microstructure on the elastic recovery properties, atomic force microscopy (AFM) was employed to characterize the surface morphology of TPAE‐*N_x_*‐*PEG_25%_* (Figure S2a). Compared to samples with other naphthalene contents, TPAE‐*N_40%_*‐*PEG_25%_* exhibits a relatively uniform microscopic surface structure, which is consistent with the observations from SEM (Figure 4d) and POM (Figure 4e) images. This homogeneous microstructure facilitates more uniform stress distribution during stretching, thereby contributing to its more stable elastic recovery behavior. The elastic recovery curves of TPAE‐*N_40%_*‐*PEG_35%_* after 10 cycles at 50% strain are shown in Figure 6g. Compared with TPAE‐*N_40%_*‐*PEG_25%_*, TPAE‐*N_40%_*‐*PEG_35%_* maintains good elastic recovery stability when the soft segment content increases to 35 mol%, with an elastic recovery ratio of 89.8% after 10 cycles, which is higher than that of TPAE‐*N_40%_*‐*PEG_25%_* (81.3%). However, when the soft segment content further increases to 50 mol%, the elastic recovery stability of TPAE‐*N_40%_*‐*PEG_50%_* decreases. As shown in Figure S2b, compared to TPAE‐*N_40%_*‐*PEG_35%_*, TPAE‐*N_40%_*‐*PEG_50%_* exhibits a less homogeneous surface morphology, resulting in weakened recovery performance stability.

The mechanical properties of TPAE‐*N_x_*‐*PEG_25%_* with different NDA contents are compared in Figure 6i. As the proportion of NDA in the molecular chains increases, the tensile strength and Young's modulus of TPAE‐*N_x_*‐*PEG_25%_* are gradually enhanced, while the fracture elongation and elastic recovery performance decrease accordingly. However, the toughness of TPAE‐*N_x_*‐*PEG_25%_* exhibits a trend of initial increase followed by a decrease with rising NDA content. For instance, when the NDA content increases from 0 to 40 mol%, the toughness significantly improves from 104.3 to 134.6 MJ/m^3^. However, as the NDA content further reaches 60 mol%, the toughness decreases to 94.3 MJ/m^3^. Figure 6j shows a comparison of the mechanical properties of TPAE‐*N_40%_*‐*PEG_y_* with different soft segment contents. With increasing soft segment content, the fracture elongation of TPAE‐*N_40%_*‐*PEG_y_* gradually increases, whereas its tensile strength, Young's modulus, and toughness all decrease.

The tension set behavior of TPAE‐*N_40%_*‐*PEG_25%_* was investigated under varying temperatures and strains, with the results summarized in Table S12. At 25°C, after maintaining a 20% tensile strain for 24 h and subsequent unloading, the sample length recovered from an initial 60.0 mm to 51.5 mm, yielding a tension set value (*E*_t_) of 15.0% as calculated by Equation S4. This low *E*_t_ is attributed to the robust physical cross‐linking network formed by the amide‐naphthalene‐amide hard segments via intermolecular hydrogen bonds. This network remains intact under 20% strain, allowing the soft segments to undergo reversible entropic deformation without irreversible damage, thus enabling efficient recovery upon unloading. In contrast, at 70°C under the same 20% strain, the recovered length was 54.8 mm, corresponding to a significantly higher *E*_t_ of 48.0%. This increase is ascribed to the enhanced molecular chain mobility and partial disruption of physical cross‐links at elevated temperature, which induces a transition from elastic to plastic behavior. When the strain was increased to 50% for 24 h, the *E*_t_ were 27.2% at 25°C and 38.0% at 70°C. The increase at 25°C (from 15.0% to 27.2%) suggests that the strain exceeds the purely elastic limit, triggering finite rearrangement of the hard segment network and introducing additional irreversible deformation. Interestingly, at 70°C, the *E*_t_ decreased from 48.0% at 20% strain to 38.0% at 50% strain. This counterintuitive trend is likely attributed to the synergistic effect of high temperature and large strain, which can potentially induce orientation rearrangement or strain‐induced crystallization of the hard segments. The formation of a relatively stable dissipative structure partially restores the material's retraction capability compared to the more disordered state at lower strain.

The compression set of TPAE‐*N_40%_*‐*PEG_25%_* was evaluated under analogous conditions, with the data presented in Table S13. At 25°C, after 24 h of 20% compression followed by pressure release, the sample height recovered from 5.08 to 6.21 mm, giving a compression set value (*C*_s_) of 14.4% via Equation S5. This excellent compression recovery is attributed to the microphase‐separated morphology, where the hard segments form a stable physical network via hydrogen bonds that effectively guides the entropic recovery of the soft segments. At 70°C under the same 20% compression, the recovered height was 6.10 mm (initial: 5.06 mm), resulting in an increased *C*_s_ of 22.4%. The elevated temperature weakens the hydrogen bonding among hard segments, reducing the network's constraint on soft segments and promoting irreversible deformation during compression. When the compression strain was increased to 50% for 24 h, the *C*_s_ were 13.9% at 25°C and 24.5% at 70°C. At 25°C, the slightly lower *C*_s_ at 50% strain (13.9%) compared to 20% strain (14.4%) indicates that larger compression promotes greater orientation of soft segments, storing higher entropic recovery energy, while the stable hard‐segment network remains intact. At 70°C, the further increase in *C*_s_ to 24.5% under 50% strain demonstrates that large deformation exacerbates the destruction and irreversible slippage of the hard‐segment network when hydrogen bonds are already weakened by heat, leading to greater accumulation of plastic deformation.

### 2.6 Flexible Printing Application and Recyclability

**FIGURE 7:**
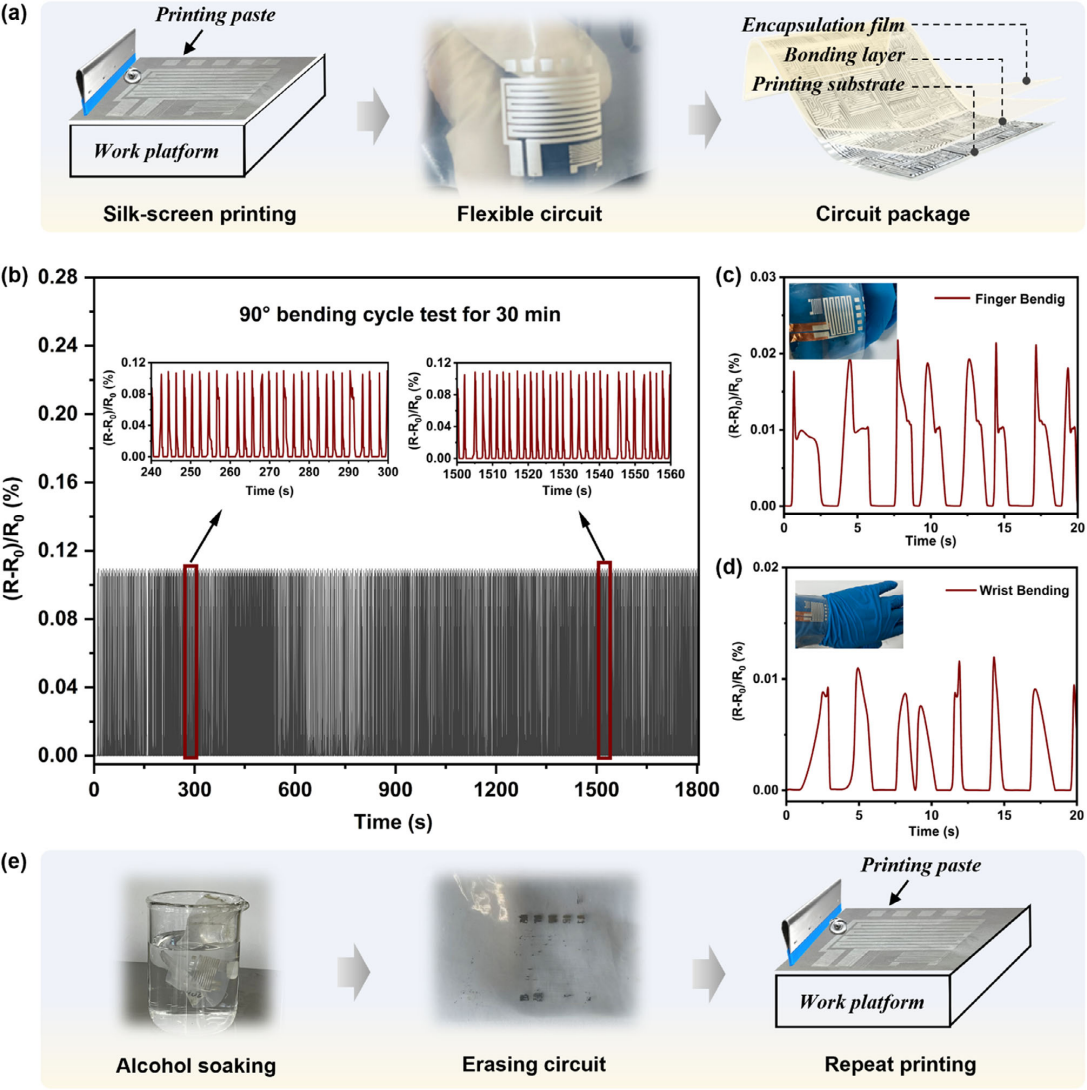
(a) TPAE*‐N_40%_‐PEG_25%_* based flexible circuit printing and packaging. (b) Flexible printed circuits maintain 0°–90°bending cycle test for 30 min to collect electrical signals. (c) Collecting finger bending signals using flexible printed circuits. (d) Collecting wrist bending signals using flexible printed circuits. (e) Recycling and reuse of flexible printing substrates.

The application of TPAE*‐N_x_‐PEG_y_* as a flexible substrate for printed electronics was investigated in this work. As illustrated in Figure 7a, a conductive circuit based on silver‐coated copper paste was fabricated on a 0.1 mm‐thick TPAE*‐N_40%_‐PEG_25%_* flexible film via silk‐screen printing technology. After printing, the circuit patterns were cured in an oven at 120°C for 3 h, followed by thin‐film encapsulation to protect the printed patterns. During the bending and recovery process, the resistance of the conductive lines printed on the flexible substrate will change accordingly: when bending, the stretching of the substrate causes the gaps in the conductive paste to increase, resulting in an increase in resistance; when recovering, the gaps decrease, and the resistance then decreases. As shown in Figure 7b, the electrical stability of the flexible printed circuit was evaluated under repeated bending conditions (0°–90°) for a duration of 30 min. The results demonstrate a stable electrical signal output throughout the cyclic bending test. Furthermore, the circuit was attached to the finger (Figure 7c) and wrist (Figure 7d), enabling effective conversion of mechanical bending into electrical signals, which suggests promising potential for applications such as smart robotics and strain sensing. Additionally, as shown in Figure 7e, the printed circuit can be completely removed by immersing the used device in alcohol for 12 h, indicating excellent recyclability and reusability of the flexible substrate.

## 3 Conclusion

In conclusion, drawing inspiration from the hierarchical architecture and dynamic sacrificial bond mechanism of spider silk, we have successfully designed and synthesized a novel class of strong and tough naphthalene‐containing thermoplastic polyamide elastomers (TPAE*‐N_x_‐PEG_y_*) via a facile and eco‐friendly melt polycondensation process. A multi‐level structure was constructed through a biomimetic design strategy within the polymer: a primary microphase separation through the alternation of rigid aliphatic polyamide and soft PEG segments, and a secondary reinforcement via the incorporation of naphthalene rings. This molecular engineering creates a robust network of physical cross‐linking points, primarily through hydrogen bonding and *π–π* stacking, which mirrors the role of β‐sheet crystals and sacrificial hydrogen bonds in spider silk. Comprehensive characterization confirmed TPAE*‐N_x_‐PEG_y_* possesses outstanding thermal properties and exceptional mechanical performance. The optimized sample, TPAE*‐N_40%_‐PEG_25%_*, achieved a remarkable balance of tensile strength (37.3 MPa), elongation at break (470.8%), toughness (134.6 MJ/m^3^), and elastic recovery (81.3%). Crucially, VT‐FTIR and 2D‐COS analysis provided deep insights into the dynamic behavior of the hydrogen‐bond network (density ranging from 41.8% to 51.7%), directly linking the energy dissipation mechanism during bond breakage and reformation to the observed enhancement in toughness and strength. A flexible substrate fabricated from TPAE*‐N_40%_‐PEG_25%_* reliably supported printed conductive circuits, exhibiting stable electrical signals under cyclic mechanical strain and accurately monitoring motions of fingers and wrists, thereby showcasing its potential for flexible strain sensors and human‐machine interfaces. Furthermore, the substrate's recyclability—achieved by simply immersing it in ethanol to remove the printed circuits—highlights its significant advantage for sustainable electronics. We are confident that this spider‐silk‐inspired molecular design strategy offers a powerful and generalizable paradigm for developing high‐performance, recyclable polymer substrates. It is paving a promising path toward the large‐scale commercialization of sustainable flexible electronic systems.

## Conflicts of Interest

The authors declare no conflicts of interest.

## Supporting information




**Supporting File 1**: advs75129‐sup‐0001‐SuppMat.docx.

## Data Availability

The data that supports the findings of this study are available in the supplementary material of this article.
