# Supplementary material for: Toward Flexible Printed Electronics: A Spider‐Silk‐Inspired, Strong and Tough Thermoplastic Polyamide Elastomer
Source: Adv Sci (Weinh). 2026 Apr 2;13(36):e75129. doi: 10.1002/advs.75129 (PMC13317553; doi:10.1002/advs.75129)
Supplement: Supplementary file 1 — Supporting File 1: advs75129‐sup‐0001‐SuppMat.docx. [file ADVS-13-e75129-s001.docx]

**Supporting Information**

**Toward Flexible Printed Electronics: A Spider-Silk-Inspired, Strong and Tough Thermoplastic** **Polyamide Elastomer**

Bo Yang^a, b^, Liyue Zhang^b^, Mengjing Yang^a, b^, Hangzhi Guo^a, b^, Zhibo Lin^c^, Xinyu Qi^c^, Wenjun Li^b^, Haiyan Yan^d^, Meng Li^d^, Yanhui Chen^b,^ *, Weixing Chen^d,^ *, Zhenguo Liu^a, b, c,^ *, Wei Huang^a, b, c^

*^a^* *Institute of Flexible Electronics, Northwestern Polytechnical University, Xi’an, 710072, China*

*^b^* *Key laboratory of Flexible Electronics of Zhejiang Province, Ningbo Institute of Northwestern Polytechnical University, 218 Qingyi Road, Ningbo, 315103, China*

*^c^ School of Flexible Electronics (SoFE) and Henan Institute of Flexible Electronics (HIFE), Henan University, 379 Mingli Road, Zhengzhou, 450046, China*

*^d^ Engineering Research Center of Light Stabilizers for Polymer Materials Universities of Shaanxi Province, School of Materials and Chemical Engineering, Xi’an Technological University, Xi’an, 710021, China*

*Corresponding authors:* [*iamzgliu@nwpu.edu.cn*](mailto:iamzgliu@nwpu.edu.cn)*;* [*chenwx@xatu.edu.cn*](mailto:chenwx@xatu.edu.cn); *yanhuichen@nwpu.edu.cn*

**1 Experimental**

**1.1 Material**

NDA (industrial grade, ≥99.9%) was purchased from Indorama Ventures Xylenes & PTA LLC (Alabama, USA). DA10 (industrial grade, ≥99%) was obtained from Shandong Dongchen Ruisen New Material Technology Co., Ltd. (Shandong, China). DDA (industrial grade, ≥99%) was supplied by China Pingmei Holdings Group Co., Ltd. (Henan, China). Polyethylene glycol with an average molecular weight of 400 (PEG-400), and trifluoroacetic acid-d were sourced from Shanghai McLean Biochemical Technology Co., Ltd. (Shanghai, China). Concentrated sulfuric acid (96%) was purchased from China National Pharmaceutical Group Chemical Reagent Co., Ltd. (Shanghai, China). All the above agents were used without further treatment.

**1.2 Synthesis of naphthalene-containing polyamide elastomer**

The NDA, DDA, DA10 and PEG-400 were added into a 2 L high-temperature and high-pressure reactor in accordance with the molar ratio of monomers as shown in Table S1 for melt polycondensation reaction. Meanwhile, 0.5 wt% of catalyst relative to the total mass of the reaction system was added into the reactor for this polycondensation reaction. The synthesis of the naphthalene-containing polyamide elastomer was performed according to the following procedure: (1) The reaction vessel was sealed and purged with nitrogen gas 10 times to displace air and establish an inert atmosphere. Subsequently, the vessel pressure was maintained at 0.2 MPa under a continuous nitrogen flow. (2) To facilitate the salt formation reaction of the naphthalene-containing polyamide elastomer hard segment and prevent volatilization of DA10, the reaction mixture was gradually heated to 80 ℃ and held at this temperature for 2 hours； (3) The temperature was increased to 220 ℃ and maintained for 4 hours to initiate the reaction between carboxyl and amino groups; (4) Low-molecular-weight byproducts generated during the reaction were slowly distilled off into a collection vessel to drive the reaction equilibrium forward; (5) After the system pressure equilibrated to atmospheric pressure, the temperature was further raised to 240 ℃ and held for 3 hours to advance the condensation reaction; (6) The system was evacuated to -0.1 MPa, the temperature was increased to 280 ℃, and maintained under these conditions for 5 hours to promote reaction completion between the soft and hard segments; (7) Following re-pressurization to 0.2 MPa with nitrogen to break the vacuum, the molten polymer was extruded from the bottom of the vessel using nitrogen pressure; (8) The extruded polymer was dried in an oven at 60 ℃ for 24 hours to yield the final product.

Table S1 The sample feeding ratio of TPAE-*N_x_*-*PEG_y_* and the quality proportion of the soft segment.

| Samples | NDA  (mol%) | DDA  (mol%) | DA10  (mol%) | PEG-400  (mol%) | Soft segment  Content (wt%) |
| --- | --- | --- | --- | --- | --- |
| TPAE-*N_0%_*-*PEG_25%_* | 0.00 | 1.00 | 0.75 | 0.25 | 21.76 |
| TPAE-*N_0%_*-*PEG_35%_* | 0.00 | 1.00 | 0.65 | 0.35 | 29.03 |
| TPAE-*N_0%_*-*PEG_50%_* | 0.00 | 1.00 | 0.50 | 0.50 | 38.72 |
| TPAE-*N_20%_*-*PEG_25%_* | 0.20 | 0.80 | 0.75 | 0.25 | 21.90 |
| TPAE-*N_20%_*-*PEG_35%_* | 0.20 | 0.80 | 0.65 | 0.35 | 29.30 |
| TPAE-*N_20%_*-*PEG_50%_* | 0.20 | 0.80 | 0. 50 | 0.50 | 38.93 |
| TPAE-*N_40%_*-*PEG_25%_* | 0.40 | 0.60 | 0.75 | 0.25 | 22.03 |
| TPAE-*N_40%_*-*PEG_35%_* | 0.40 | 0.60 | 0.65 | 0.35 | 29.37 |
| TPAE-*N_40%_*-*PEG_50%_* | 0.40 | 0.60 | 0. 50 | 0.50 | 39.15 |
| TPAE-*N_60%_*-*PEG_25%_* | 0.60 | 0.40 | 0.75 | 0.25 | 22.17 |
| TPAE-*N_60%_*-*PEG_35%_* | 0.60 | 0.40 | 0.65 | 0.35 | 29.55 |
| TPAE-*N_60%_*-*PEG_50%_* | 0.60 | 0.40 | 0.50 | 0.50 | 39.37 |

**1.3 Characterization**

The characterization methods were listed in the Supporting Information.

FT-IR was measured using ATR mode on Bruker INVENIO-S, and FT-IR spectra were obtained by scanning in the range of 4000-400 cm^-1^ with a resolution of 4 cm^-1^. Situ infrared spectroscopy was used to characterize the temperature-dependent shifts of the C=O amide bond peaks for TPAE*-N_x_-PEG_y_* from 30 to 150 ℃. Trifluoroacetic acid-d was used as the solvent, ^1^H NMR was recorded at Bruker AVANCE NEO 500M.

A single sample was dissolved in concentrated sulfuric acid (96%) to obtain a solution of 1 g/dL, and then the relative viscosity (*η*_r_) of the polymer was measured on the HSY-4182 relative viscosity tester (eq S1), and the temperature of the water bath was set at 25±0.02 ℃. The increased viscosity *η*_sp_ (eq S2) of the polymer was calculated using the relative viscosity.

| $\text{η}_{\text{r}}=\text{t/}\text{t}_{\text{0}}$ | (eq S1) |
| --- | --- |
| $\text{η}_{\text{sp}}\text{=}\text{η}_{\text{r}}\text{-1}$ | (eq S2) |

In the above formula, *t*_0_ and *t* represent the flow time of the solvent (concentrated sulfuric acid) and the solution.

Gel Permeation Chromatography (GPC) was performed using an American Agilent GPC 50. The mobile phase was hexafluoroisopropanol (HFIP) at a temperature of 35 ℃ and a flow rate of 1 mL/min. The poly (methyl methacrylate) (PMMA) standards were used to determine molecular weights and dispersity (Đ).

The true density of the TPAE-*N_x_*-*PEG_y_* was tested on the Microtrac MRB BELPYCNO true density meter. The powder polymer was filled two-thirds of the way into the test chamber. Inert helium gas was used to fill the gaps between the powder particles. The true density of the polymer was tested at 25±0.01 ℃.

The Wide-angle X-ray diffraction (WAXD) patterns were collected on a Bruker D8 focus instrument using Cu-Ka radiation in the 2θ scan range of 5° to 60° with a scanning speed of 0.1 s per step. The crystallinity (*X*_c_) of TPAE-*N_x_*-*PEG_y_* was calculated using the WAXD peak area integration method according to the following formula (eq S3):

| *X_c_* = [(*I*-*I_a_*)/*I*]*100% | (eq S3) |
| --- | --- |

where *I* is the integrated area of the entire WAXD pattern, and *I*_a_ corresponds to the amorphous region integration area.

The DSC curves were measured on Netsch DSC 200 F3 in an N_2_ atmosphere. The DSC curves were obtained by taking a 5 mg sample and conducting a cycle test of heating, cooling, and heating at a rate of 10 ℃/min between -70 ℃ and 300 ℃, and holding the sample for 5 min when the temperature first rose to 300 ℃ and when the temperature dropped to -70 ℃.

The test curves of the TGA were obtained on the METTLER TOLEDO TGA/DSC 3+1100 LF. The 2 mg sample was placed in an alumina crucible and tested under an N_2_ atmosphere at a rate of 10 ℃/min from 30 ℃ to 600 ℃.

On the dynamic thermomechanical analyzer of NETZSCH-DMA 242E model, DMA test was carried out from -100 ℃ to 100 ℃ under shear mode at a frequency of 1 Hz and a heating rate of 3 ℃/min, with a displacement amplitude of 3 μm. The rectangular sample with the size of 18×4× 0.1 mm^3^ was prepared by the hot-pressing method for testing.

The scanning electron microscope (SEM, TESCAN SOLARIS X) with an acceleration voltage of 15 kV was used to characterize the morphology of TPAE-*N_x_*-*PEG_y_*. All samples were covered with platinum in vacuum through sputtering to avoid charging. The phase distribution of TPAE-*N_x_*-*PEG_y_* was observed using atomic force microscopy (AFM, MultiMode8, Germany). The crystal morphology of the sample was observed by using an Olympus BX61 polarized optical microscope (POM). The sample was placed in the middle of a transparent glass sheet, heated, and melted to eliminate thermal history, and then subjected to a certain pressure and naturally cooled to room temperature, resulting in a thin film with a thickness of 100 μm.

Long strip samples were prepared for testing by the hot-pressing molding method. Tensile measurements were performed according to Chinese standard GB/T 1040.3–2006 on an electronic universal testing machine (UTM4104X) at a rate of 50 mm/min. The data of mechanical properties were averaged from five specimens (50 mm × 10 mm × 1 mm). Cyclic tensile tests were carried out using the same universal testing machine at a strain rate of 5 mm/min and a 20 mm initial grip distance, and the dimensions of samples were 50 mm × 10 mm × 1 mm.

The tension set test was conducted on an electronic universal testing machine (UTM4104X) in accordance with the Chinese standard GB/T 42279-2022. The specimen dimensions were 50 mm × 6 mm × 2 mm, with a tensile speed of 50 mm/min and a tensile holding time of 24 h. Temperature control during the tension set test was achieved by means of a temperature chamber attached to the electronic universal testing machine. The tension set of TPAE-*N_x_*-*PEG_y_* was calculated according to eq S4:

| *E_t_* = [(*L_2_* - *L_0_*)/(*L_1_* - *L_0_*)]**100%* | (eq S4) |
| --- | --- |

where *L_0_* denotes the reference length of the specimen before stretching, *L_1_* denotes the length of the specimen when the preset strain is reached, *L_2_* denotes the length of the specimen after 30 min of stress release, and *E_t_* is the tension set of the specimen.

The compression set test was performed on an electronic universal testing machine (UTM4104X) following the Chinese standard GB/T 7759.1-2015. The specimen was a cylinder with a diameter of 13 mm and a thickness of 6 mm (composed of three 2 mm thick layers). The compression speed was set at 50 mm/min, and the compression holding time was 22 h. Temperature control during the test was achieved by means of a temperature chamber attached to the electronic universal testing machine. The compression set of TPAE-*N_x_*-*PEG_y_* was calculated according to eq S5:

| *C_s_* = [(*H_0_* - *H_2_*)/(*H_0_* - *H_1_*)]**100%* | (eq S5) |
| --- | --- |

where *H_0_* denotes the initial height of the specimen before compression, *H_1_* denotes the height of the specimen when the preset compressive deformation is reached, *H_2_* denotes the height of the specimen after releasing the compressive stress for 30 min, and *C_s_* represents the compression set of the specimen.

**2 Results and Discussion**

Table S2 The Relative Viscosity, Increased Viscosity, and Inherent Viscosity of TPAE-*N_x_*-*PEG_y_*.

| **Samples** | ***ŋ*_r_** | ***ŋ*_sp_** | **[*ŋ*_int_] (dL/g)** |
| --- | --- | --- | --- |
| TPAE-*N_0%_*-*PEG_25%_* | 2.2769 | 1.2769 | 0.9530 |
| TPAE-*N_0%_*-*PEG_35%_* | 2.1734 | 1.1734 | 0.8912 |
| TPAE-*N_0%_*-*PEG_50%_* | 2.1498 | 1.1498 | 0.8768 |
| TPAE-*N_20%_*-*PEG_25%_* | 2.2517 | 1.2517 | 0.9381 |
| TPAE-*N_20%_*-*PEG_35%_* | 2.2091 | 1.2091 | 0.9127 |
| TPAE-*N_20%_*-*PEG_50%_* | 2.1693 | 1.1693 | 0.8887 |
| TPAE-*N_40%_*-*PEG_25%_* | 2.3128 | 1.3128 | 0.9740 |
| TPAE-*N_40%_*-*PEG_35%_* | 2.2176 | 1.2176 | 0.9178 |
| TPAE-*N_40%_*-*PEG_50%_* | 2.1954 | 1.1954 | 0.9044 |
| TPAE-*N_60%_*-*PEG_25%_* | 2.2502 | 1.2502 | 0.9375 |
| TPAE-*N_60%_*-*PEG_35%_* | 2.2463 | 1.2463 | 0.9349 |
| TPAE-*N_60%_*-*PEG_50%_* | 2.1536 | 1.1536 | 0.8792 |

Table S3 The *T*_c_, *T_m_*, *T_g_*, *T_d, 5%_* and *T_d, max_* of TPAE-*N_x_*-*PEG_y_*.

| Samples | *T*_c1_  (℃) | *T*_c2_  (℃) | *T*_m1_  (℃) | *T*_m2_  (℃) | *T*_g1_  (℃) | *T*_g2_  (℃) | *T*_d, 5%_  (℃) | *T*_d, max_  (℃) |
| --- | --- | --- | --- | --- | --- | --- | --- | --- |
| TPAE-*N_0%_*-*PEG_25%_* | 141.4 | / | 173.4 | / | -35.9 | 58.5 | 389.0 | 442.6/463.2 |
| TPAE-*N_0%_*-*PEG_35%_* | 147.5 | / | 170.6 | / | / | / | / | / |
| TPAE-*N_0%_*-*PEG_50%_* | 136.9 | / | 158.1 | / | / | / | / | / |
| TPAE-*N_20%_*-*PEG_25%_* | 132.5 | / | 162.1 | / | -19.0 | 57.7 | 399.7 | 434.0/451.0/464.8 |
| TPAE-*N_20%_*-*PEG_35%_* | 129.4 | / | 155.5 | / | / | / | / | / |
| TPAE-*N_20%_*-*PEG_50%_* | 120.3 | / | 146.8 | / | / | / | / | / |
| TPAE-*N_40%_*-*PEG_25%_* | 117.5 | 172.6 | 137.5 | 192.7 | -12.1 | 55.6 | 398.1 | 439.6/465.4 |
| TPAE-*N_40%_*-*PEG_35%_* | 112.6 | 162.6 | 133.5 | 186.1 | -20.7 | 55.6 | 392.6 | 441.2 |
| TPAE-*N_40%_*-*PEG_50%_* | 103.7 | 153.5 | 124.9 | 177.4 | -28.0 | / | 391.0 | 440.6 |
| TPAE-*N_60%_*-*PEG_25%_* | / | 184.6 | / | 230.6 | 1.7 | 58.1 | 394.2 | 435.0/468.6 |
| TPAE-*N_60%_*-*PEG_35%_* | / | 185.6 | / | 227.5 | / | / | / | / |
| TPAE-*N_60%_*-*PEG_50%_* | / | 169.3 | / | 195.7 | / | / | / | / |

Table S4 Summary of the assignment of the deconvoluted subpeaks in the FT-IR C=O absorption bands for the TPAE*-N_0%_-PEG_25%_*, TPAE*-N_20%_-PEG_25%_*, TPAE*-N_40%_-PEG_25%_*, TPAE*-N_60%_-PEG_25%_*.

| Assignment | Sub  peak | Wavenumber (cm^-1^) | | | | Area (%) | | | |
| --- | --- | --- | --- | --- | --- | --- | --- | --- | --- |
|  |  | TPAE*-N_0%_-PEG_25%_* | TPAE*-N_20%_-PEG_25%_* | TPAE*-N_40%_-PEG_25%_* | TPAE*-N_60%_-PEG_25%_* | TPAE*-N_0%_-PEG_25%_* | TPAE*-N_20%_-PEG_25%_* | TPAE*-N_40%_-PEG_25%_* | TPAE*-N_60%_-PEG_25%_* |
| amideυ(C=O) | Free | I  (1640) | I  (1642) | I  (1640) | I  (1639) | 42.5 | 43.5 | 51.3 | 43.7 |
|  | H-bonded  (Disordered) | II  (1634) | II  (1634) | II  (1627) | II  (1627) | 29.4 | 27.2 | 18.0 | 21.9 |
|  | H-bonded  (Ordered) | III  (1613) | III  (1611) | III  (1598) | III  (1597) | 6.0 | 1.3 | 5.5 | 8.3 |
| esterυ(C=O) | Free | IV  (1735) | IV  (1732) | IV  (1738) | IV  (1739) | 14.9 | 14.3 | 6.9 | 4.6 |
|  | H-bonded  (Ordered) | V  (1721) | V  (1724) | V  (1720) | V  (1720) | 7.2 | 13.7 | 18.3 | 21.5 |
| Total degree of H-bonded | / | | | | | 42.6 | 42.2 | 41.8 | 51.7 |

Table S5 The sign of cross-peaks in synchronous spectrum of TPAE-*N_40%_*-*PEG_25%_*.

| 1598 cm^-1^ | - | - | + | + |  |
| --- | --- | --- | --- | --- | --- |
| 1627 cm^-1^ | - | + | + |  |  |
| 1640 cm^-1^ | - | + |  |  |  |
| 1720 cm^-1^ | - |  |  |  |  |
| 1738 cm^-1^ |  |  |  |  |  |
|  | 1738 cm^-1^ | 1720 cm^-1^ | 1640 cm^-1^ | 1627 cm^-1^ | 1598 cm^-1^ |

Table S6 The sign of cross-peaks in asynchronous spectrum of TPAE-*N_40%_*-*PEG_25%_*.

| 1598 cm^-1^ | + | + | - | - |  |
| --- | --- | --- | --- | --- | --- |
| 1627 cm^-1^ | - | + | - |  |  |
| 1640 cm^-1^ | - | + |  |  |  |
| 1720 cm^-1^ | + |  |  |  |  |
| 1738 cm^-1^ |  |  |  |  |  |
|  | 1738 cm^-1^ | 1720 cm^-1^ | 1640 cm^-1^ | 1627 cm^-1^ | 1598 cm^-1^ |

Table S7 The results of multiplication on the sign of each cross-peaks in synchronous and asynchronous spectrum of TPAE-*N_40%_*-*PEG_25%_*.

| 1598 cm^-1^ | - | - | - | - |  |
| --- | --- | --- | --- | --- | --- |
| 1627 cm^-1^ | + | + | - |  |  |
| 1640 cm^-1^ | + | + |  |  |  |
| 1720 cm^-1^ | - |  |  |  |  |
| 1738 cm^-1^ |  |  |  |  |  |
|  | 1738 cm^-1^ | 1720 cm^-1^ | 1640 cm^-1^ | 1627 cm^-1^ | 1598 cm^-1^ |

1598 cm^-1^→1720 cm^-1^→1738 cm^-1^→1640 cm^-1^→1627 cm^-1^

**
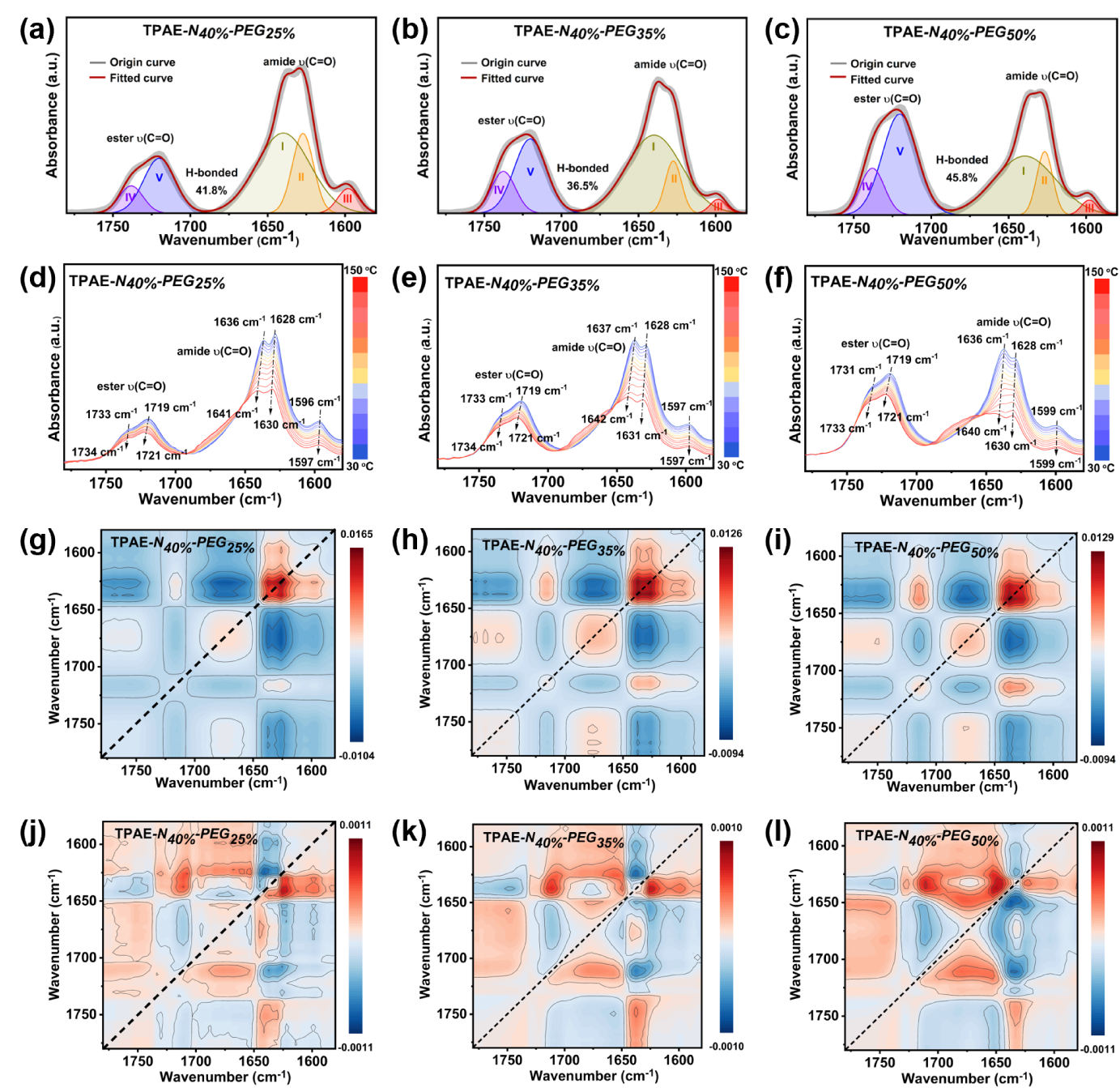
**

Figure S1 FTIR spectra of (a) TPAE-*N_40%_*-*PEG_25%_*, (b) TPAE-*N_40%_*-*PEG_35%_*, (c) TPAE-*N_40%_*-*PEG_50%_* in the C=O stretching vibration region. VT-FTIR spectra of (d) TPAE-*N_40%_*-*PEG_25%_*, (e) TPAE-*N_40%_*-*PEG_35%_*, (f) TPAE-*N_40%_*-*PEG_50%_* upon heating from 30 ℃ to 150 ℃ in the range of 1580-1780 cm^-1^. The 2D-COS synchronous map of (g) TPAE-*N_40%_*-*PEG_25%_*, (h) TPAE-*N_40%_*-*PEG_35%_*, (i) TPAE-*N_40%_*-*PEG_50%_* under heating condition from 30 ℃ to 150 ℃ in the range of 1580-1780 cm^-1^. The 2D-COS asynchronous map of (j) TPAE-*N_40%_*-*PEG_25%_*, (k) TPAE-*N_40%_*-*PEG_35%_*, (l) TPAE-*N_40%_*-*PEG_50%_* under heating condition from 30 ℃ to 150 ℃ in the range of 1580-1780 cm^-1^.

Table S8 Summary of the assignment of the deconvoluted subpeaks in the FT-IR C=O absorption bands for the TPAE*-N_40%_-PEG_25%_*, TPAE*-N_40%_-PEG_35%_*, TPAE*-N_40%_-PEG_50%_*.

| Assignment | Subpeak | Wavenumber (cm^-1^) | | | Area (%) | | |
| --- | --- | --- | --- | --- | --- | --- | --- |
|  |  | TPAE*-N_40%_-PEG_25%_* | TPAE*-N_40%_-PEG_35%_* | TPAE*-N_40%_-PEG_50%_* | TPAE*-N_40%_-PEG_25%_* | TPAE*-N_40%_-PEG_35%_* | TPAE*-N_40%_-PEG_50%_* |
| amideυ(C=O) | Free (I) | I (1640) | I (1640) | I (1640) | 51.3 | 52.8 | 43.4 |
|  | H-bonded  (II) | II (1627) | II (1627) | II (1627) | 18.0 | 8.9 | 9.8 |
|  | H-bonded  (III) | III (1598) | III (1598) | III (1598) | 5.5 | 2.4 | 2.3 |
| esterυ(C=O) | Free (IV) | IV (1738) | IV (1738) | IV (1738) | 6.9 | 10.7 | 10.8 |
|  | H-bonded  (V) | V (1720) | V (1720) | V (1720) | 18.3 | 25.2 | 33.7 |
| Total degree of H-bonded | / | | | | 41.8 | 36.5 | 45.8 |

Table S9 The sign of cross-peaks in synchronous spectrum of TPAE-N40%-PEG50%.

| 1598 cm^-1^ | - | + | + | + |  |
| --- | --- | --- | --- | --- | --- |
| 1627 cm^-1^ | - | + | + |  |  |
| 1640 cm^-1^ | - | + |  |  |  |
| 1720 cm^-1^ | - |  |  |  |  |
| 1738 cm^-1^ |  |  |  |  |  |
|  | 1738 cm^-1^ | 1720 cm^-1^ | 1640 cm^-1^ | 1627 cm^-1^ | 1598 cm^-1^ |

Table S10 The sign of cross-peaks in asynchronous spectrum of TPAE-*N_40%_*-*PEG_50%_*.

| 1598 cm^-1^ | - | - | - | - |  |
| --- | --- | --- | --- | --- | --- |
| 1627 cm^-1^ | - | + | + |  |  |
| 1640 cm^-1^ | - | + |  |  |  |
| 1720 cm^-1^ | - |  |  |  |  |
| 1738 cm^-1^ |  |  |  |  |  |
|  | 1738 cm^-1^ | 1720 cm^-1^ | 1640 cm^-1^ | 1627 cm^-1^ | 1598 cm^-1^ |

Table S11 The results of multiplication on the sign of each cross-peaks in synchronous and asynchronous spectrum of TPAE-*N_40%_*-*PEG_50%_*.

| 1598 cm^-1^ | + | - | - | - |  |
| --- | --- | --- | --- | --- | --- |
| 1627 cm^-1^ | + | + | + |  |  |
| 1640 cm^-1^ | + | + |  |  |  |
| 1720 cm^-1^ | + |  |  |  |  |
| 1738 cm^-1^ | + |  |  |  |  |
|  | 1738 cm^-1^ | 1720 cm^-1^ | 1640 cm^-1^ | 1627 cm^-1^ | 1598 cm^-1^ |

1738 cm^-1^→1598 cm^-1^→1720 cm^-1^→1640 cm^-1^→1627 cm^-1^

Table S12 Tension set of TPAE*-N_40%_-PEG_25%_* at 25 ℃ and 70 ℃.

| Length | Tension set at 25 ℃ | | Tension set at 70 ℃ | |
| --- | --- | --- | --- | --- |
|  | 20% | 50% | 20% | 50% |
| *L_0_* (mm) | 50.0 | 50.0 | 50.0 | 50.0 |
| *L_1_* (mm) | 60.0 | 75.0 | 60.0 | 75.0 |
| *L_2_* (mm) | 51.5 | 56.8 | 54.8 | 59.5 |
| *E_t_* (%) | 15.0 | 27.2 | 48.0 | 38.0 |

Table S13 Compression set of TPAE*-N_40%_-PEG_25%_* at 25 ℃ and 70 ℃.

| Height | Compression set at 25 ℃ | | Compression set at 70 ℃ | |
| --- | --- | --- | --- | --- |
|  | 20% | 50% | 20% | 50% |
| *H_0_* (mm) | 6.40 | 6.40 | 6.40 | 6.40 |
| *H_1_* (mm) | 5.08 | 3.24 | 5.06 | 3.26 |
| *H_2_* (mm) | 6.21 | 5.96 | 6.10 | 5.63 |
| *C_s_* (%) | 14.4 | 13.9 | 22.4 | 24.5 |

**
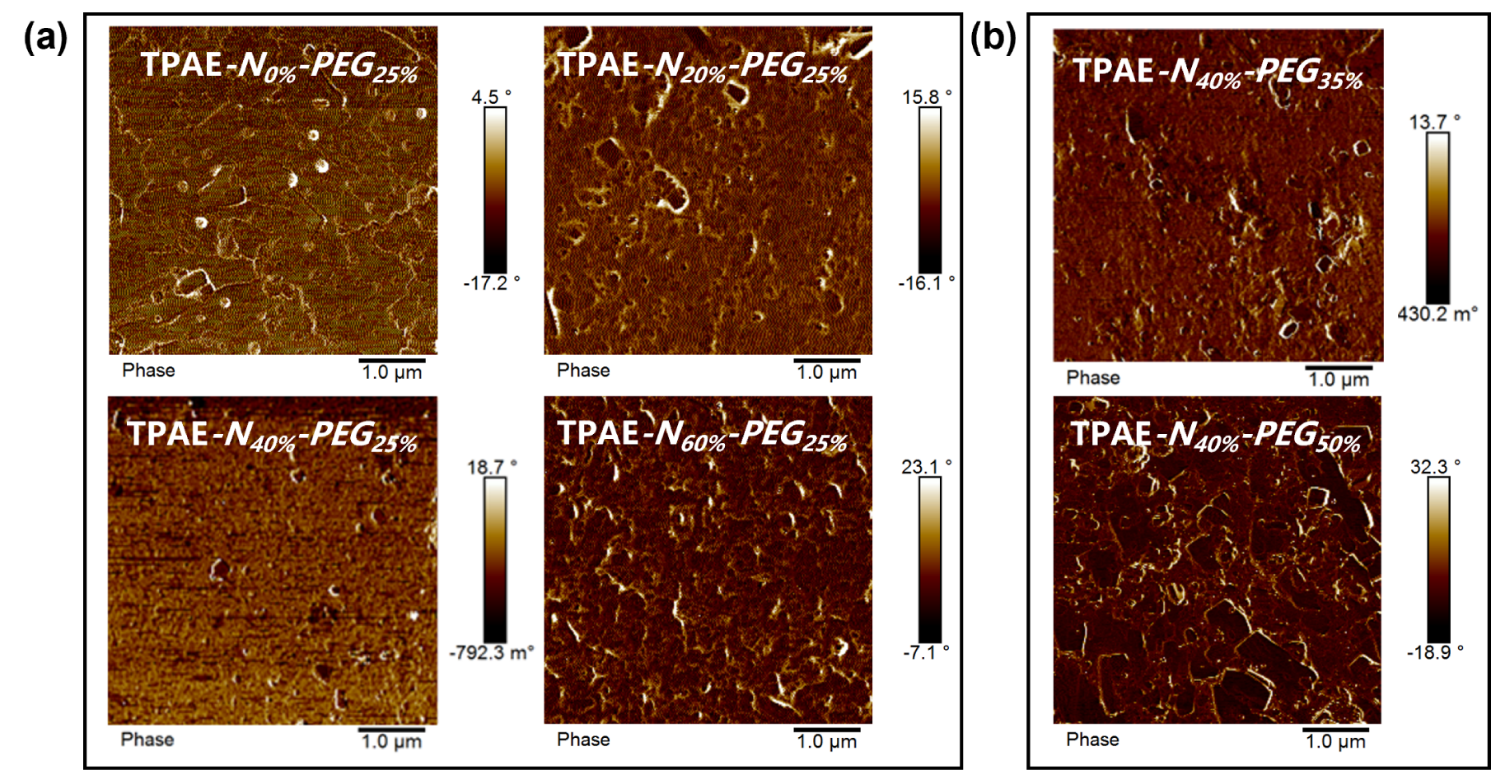
**

Figure S2 (a) AFM phase images of TPAE-*N_x_*-*PEG_25%_*. (b) AFM phase images of TPAE-*N_40%_*-*PEG_y_*.
